# Two stress-responsive kinases suppress ferroptosis by activating antioxidant programs under mild oxidative stress

**DOI:** 10.1038/s41392-026-02892-1

**Published:** 2026-08-03

**Authors:** Yumiko Fujikawa, Hirotatsu Imai, Tetsuo Onuki, Kouji Hoshino, Marco A. De Velasco, Kazuhiko Matsuo, Hitomi Kurosawa, Kyoko Aoyagi, Hiroko Hirose, Yoshie Nakamura, Akiko Uchiyama, Kae Suzuki, Mariko Mizuguchi, Hidehisa Takahashi, Hiroyuki Osada, Noritaka Kagaya, Kazuo Shin-ya, Hiroyuki Satofuka, Yukinari Kato, Hidehito Kuroyanagi, Daisuke Utsumi, Kenzo Takahashi, Takashi Nakayama, Hirotsugu Uemura, Hiroji Uemura, Minoru Yoshida, Shigeo Ohno, Akio Yamashita

**Affiliations:** 1 Department of Investigative Medicine, University of the Ryukyus Graduate School of Medicine, Ginowan, Okinawa Japan; 2 Laboratory of Cancer Biology, Institute for Diseases of Old Age, Juntendo University Graduate School of Medicine, Hongo, Tokyo Japan; 3 Department of Biochemistry, University of the Ryukyus Graduate School of Medicine, Ginowan, Okinawa Japan; 4 Chemical Genomics Research Group, RIKEN Center for Sustainable Resource Science, Wako, Saitama Japan; 5 Department of Urology & Renal Transplantation, Yokohama City University Medical Center, Yokohama, Kanagawa Japan; 6 Department of Genome Biology, Kindai University Faculty of Medicine, Sakai, Osaka Japan; 7 Division of Chemotherapy, Kindai University Faculty of Pharmacy, Higashi-Osaka, Osaka Japan; 8 Department of Molecular Cell Biology, Yokohama City University Graduate School of Medicine, Yokohama, Kanagawa Japan; 9 Department of Molecular Biology, Yokohama City University Graduate School of Medicine, Yokohama, Kanagawa Japan; 10 Laboratory of Immunology, Department of Medical Technology, Azabu University School of Life and Environmental Science, Sagamihara, Kanagawa Japan; 11 Chemical Resource Development Unit and Natural Product Depository (NPDepo), Wako, Saitama Japan; 12 Institute of Microbial Chemistry (BIKAKEN), Shinagawa-ku, Tokyo Japan; 13 Technology Research Association for Next Generation Natural Products Chemistry; Aomi, Koto-ku, Tokyo Japan; 14 National Institute of Advanced Industrial Science and Technology (AIST); Aomi, Koto-ku, Tokyo Japan; 15 Department of Antibody Drug Development, Tohoku University Graduate School of Medicine, Sendai, Miyagi Japan; 16 Advanced Medical Research Center, University of the Ryukyus, Ginowan, Okinawa Japan; 17 Department of Dermatology, University of the Ryukyus Graduate School of Medicine, Ginowan, Okinawa Japan; 18 Department of Innovative Medicine, Kindai University Faculty of Medicine, Sakai, Osaka Japan; 19 Drug Discovery Seeds Development Unit, RIKEN Center for Sustainable Resource Science, Wako, Saitama Japan; 20 Office of University Professors, The University of Tokyo; Yayoi, Bunkyo-ku, Tokyo Japan; 21 Division of Molecular Cell Biology, Kindai University Faculty of Pharmacy, Higashi-Osaka, Osaka Japan

**Keywords:** Biochemistry, Drug discovery, Cancer metabolism, Cell biology

## Abstract

Cancer cells maintain chronically elevated levels of reactive oxygen species (ROS) while relying on robust antioxidant programs to preserve redox homeostasis and viability. Although therapeutic strategies that disrupt this balance to induce lethal oxidative stress and ferroptosis have emerged as promising anticancer approaches, the upstream signaling mechanisms that constrain ROS accumulation under physiologically relevant stress conditions remain incompletely understood. Here, we identify the stress-responsive kinases SMG1 and DNA-dependent protein kinase (DNA-PK) as functionally redundant regulators of redox homeostasis and ferroptosis resistance. Genetic or pharmacological inhibition of either kinase triggers ferroptotic cell death, accompanied by marked accumulation of total ROS, ferrous iron, and lipid hydroperoxides. Mechanistically, under mild oxidative stress, SMG1 and DNA-PK cooperatively phosphorylate the central antioxidant transcription factor NRF2 at serine 13 and serine 40, weakening its interaction with the negative regulator KEAP1 and promoting NRF2 accumulation and transcriptional activation. Transcriptomic profiling of de novo mRNAs revealed that inhibition of either kinase is sufficient to suppress NRF2-driven antioxidant gene expression. In contrast, excessive oxidative stress overrides this pro-survival pathway and redirects signaling toward anti-survival responses mediated by ATF4, ATM–CHK2, and JNK/p38 pathways. Collectively, these findings uncover a previously unrecognized SMG1/DNA-PK–NRF2 signaling axis that functions as a redox stress–intensity–dependent switch governing cell fate decisions between antioxidant adaptation and ferroptotic death. Targeting this axis may represent a promising therapeutic strategy for cancer treatment.

## Introduction

Cells maintain redox homeostasis through tightly coordinated systems that balance intracellular production and elimination of reactive oxygen species (ROS). In cancer cells, metabolic reprogramming—including enhanced oxidative phosphorylation, altered redox enzyme activity, and increased metal ion flux—results in chronically elevated basal ROS levels. To survive under these conditions, cancer cells augment antioxidant capacity, thereby sustaining proliferation while remaining highly sensitive to perturbations in redox balance.^1^ This adaptive state renders cancer cells particularly vulnerable to disruptions in ROS homeostasis, positioning redox regulation as an attractive therapeutic target.

Disruption of redox homeostasis initially elicits adaptive pro-survival responses that enable cells to tolerate and recover from mild oxidative stress. In contrast, sustained or excessive ROS accumulation overwhelms antioxidant capacity and activates cell death pathways, including apoptosis and ferroptosis.^2^ Accordingly, the ability of cells to discriminate between mild and excessive oxidative stress and to engage appropriate downstream signaling axes is a critical determinant of cell fate.^2^ Despite extensive characterization of redox-regulated pathways, the molecular mechanisms that selectively activate pro-survival signaling in response to mild oxidative stress remain incompletely understood.

Ferroptosis is a distinct, non-apoptotic form of regulated cell death driven by iron-dependent, or iron-amplified, accumulation of lipid hydroperoxides (LOOHs) resulting from dysregulated intracellular ROS homeostasis.^3^ Excess superoxide anion (O^2−^·), predominantly generated by the mitochondrial electron transport chain, is rapidly dismutated to hydrogen peroxide (H_2_O_2_). When not adequately detoxified, H_2_O_2_ promotes lipid peroxidation through iron-catalyzed reactions, generating lipid radicals that damage cellular membranes and drive LOOH accumulation, thereby executing ferroptotic cell death.^3^ Beyond cancer, ferroptosis contributes to the pathogenesis of diverse diseases, including acute kidney injury, neurodegeneration, cardiovascular disorders, and liver disease.^4–6^

A central regulator of antioxidant defense and ferroptosis resistance is NF-E2-related factor 2 (NRF2).^7,8^ NRF2 suppresses ferroptosis through transcriptional induction of genes involved in glutathione metabolism, cystine uptake, lipid peroxide detoxification, and heme metabolism, including SLC7A11, GCLC/GCLM, GPXs, and HO-1.^9^ Consistent with its cytoprotective function, constitutive NRF2 activation is frequently observed in human cancers, where it supports tumor growth and therapy resistance.^10^

Under basal conditions, NRF2 protein levels are tightly controlled by ubiquitin-dependent proteasomal degradation mediated by multiple E3 ubiquitin ligase adaptor systems.^11,12^ Among them, KEAP1 is the most extensively characterized regulator; oxidation of specific cysteine residues within KEAP1 inhibits NRF2 ubiquitination, allowing NRF2 stabilization and transcriptional activation of genes involved in redox metabolism, detoxification, and ferroptosis resistance during oxidative stress.^13–16^ In addition to redox-dependent regulation, phosphorylation of NRF2 at serine 40 (NRF2-S40) has been proposed to modulate KEAP1 binding. However, the identity of the responsible kinase(s) and the physiological relevance of this modification remain controversial.^17,18^ Notably, large-scale kinase-substrate analyses indicate that NRF2-S40 does not conform to canonical protein kinase C (PKC) motifs, raising the possibility that non-canonical kinases mediate NRF2 phosphorylation.^19^

Suppressor with morphogenetic effect on genitalia 1 (SMG1) and DNA-dependent protein kinase catalytic subunit (DNA-PKcs) belong to the phosphatidylinositol 3-kinase-related protein kinase (PIKK) family, which also includes mammalian target of rapamycin (mTOR), ataxia telangiectasia mutated (ATM), ATM- and Rad3-related (ATR). Except for mTOR, PIKKs share substrate specificity for serine/threonine–glutamine (S/TQ) motifs and function as central regulators of cellular stress responses. Although PIKKs have been implicated in oxidative stress signaling and ferroptosis regulation, whether and how individual PIKKs directly regulate NRF2 remains unclear.^20–27^

SMG1 is best characterized as a core kinase in nonsense-mediated mRNA decay (NMD) through phosphorylation of UPF1^28^; however, its functions beyond NMD, including potential roles in oxidative stress responses, remain only partially defined.^20,29^ Similarly, DNA-PK has been implicated in redox-associated stress responses, suggesting the possibility of functional cooperation or redundancy among PIKKs in controlling antioxidant pathways.^25^

Here, we demonstrate that mild oxidative stress selectively activates an SMG1– and DNA-PK–dependent NRF2 signaling axis. Using pharmacological and genetic approaches, we show that SMG1 and DNA-PK directly phosphorylate NRF2 at Ser-13 and Ser-40, residues proximal to the DLG motif required for KEAP1 binding. These phosphorylation events weaken NRF2-KEAP1 association, promote NRF2 accumulation, and induce antioxidant gene expression without suppressing nonsense-mediated mRNA decay. Inhibition of SMG1 and DNA-PK disrupts redox homeostasis, resulting in increased ROS levels and ferrous iron accumulation, lipid hydroperoxide generation, reduced cell viability, and enhanced ferroptotic vulnerability in cancer cells and mouse liver. Collectively, our findings uncover a previously unrecognized SMG1/DNA-PK–NRF2 axis that senses mild oxidative stress to maintain redox homeostasis and promote cell survival, providing new mechanistic insight into adaptive antioxidant signaling in cancer.

## Results

### A specific inhibitor of SMG1 is identified and characterized

Because SMG1 is an essential component of the NMD surveillance machinery, knockdown of SMG1 causes stabilization of NMD substrate mRNAs, including those encoding stress-responsive transcription factors such as ATF4. Accumulation of these substrates can pre-activate downstream stress-response programs, confounding the interpretation of stress signaling.^30–32^ To address this limitation, we sought to identify a selective pharmacological inhibitor of SMG1 kinase activity. To this end, we established an AlphaScreen-based in vitro SMG1 kinase assay system and validated its performance using caffeine, a known SMG1 inhibitor (Supplementary Fig. 1a, b). Using this platform, we screened chemical libraries comprising 46,560 compounds and identified 219 molecules that exhibited 50–80% inhibition of SMG1 kinase activity. Among these, 38 compounds displayed half-maximal inhibitory concentrations (IC_50_) below 1 μM. To further validate SMG1 inhibition, we directly measured kinase activity using [γ-^32^P]ATP. Of the 38 candidates, 11 compounds showed clear inhibitory activity against SMG1 in this assay. Among these, five compounds exhibited preferential inhibitory activity toward SMG1 relative to other kinase-active members of the PIKK family (Supplementary Table 1).

**Fig. 1:**
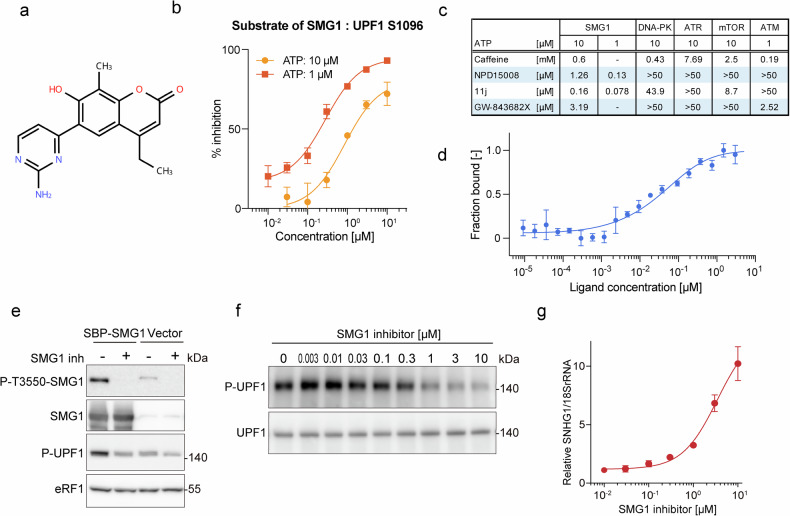
Discovery of a selective SMG1 inhibitor enables systematic dissection of SMG1 signaling. **a** Chemical structure of NPD15008. **b** Inhibitory kinetics of NPD15008 against SMG1 kinase activity. In vitro SMG1 activity toward a peptide substrate was measured and plotted as percentage inhibition versus the indicated concentrations of NPD15008 in the presence of 10 μM (circles) or 1 μM (squares) nonradiolabeled ATP. All reactions were performed in the presence of 16.6 nM [γ-^32^P]ATP. Details are described in the Materials and Methods. Data represent the mean ± standard error. **c** Comparison of IC₅₀ values to assess the specificity of SMG1 inhibition. In vitro kinase activities of PIKKs (SMG1, DNA-PK, ATR, mTOR, and ATM) toward peptide substrates were measured in the presence of the indicated compounds using [γ-^32^P]ATP and nonradiolabeled ATP at the indicated concentrations. Details are described in the “Materials and Methods”. Data represent the mean ± standard error of IC₅₀ values from three independent experiments. **d** Microscale thermophoresis (MST) analysis of the interaction between NPD15008 and the SMG1–SMG9 complex. Binding curves were generated based on changes in thermophoretic mobility upon titration of NPD15008 to fluorescently labeled SMG1. Data represent the mean ± standard error of *K*_*D*_ values from two independent experiments. **e** Effect of NPD15008 on SMG1 autophosphorylation at T3550. HEK293T cells were transfected with SBP-SMG1 or an empty vector. Forty-four hours after transfection, cells were treated with 6 μM NPD15008 for 4 h. Whole-cell lysates were analyzed by immunoblotting with the indicated antibodies. **f** Inhibition of UPF1 phosphorylation by NPD15008 in cells. HeLa Tet-Off cells were treated with serial dilutions of NPD15008 for 2 h. Whole-cell lysates were analyzed by immunoblotting using antibodies against phospho-S1096-UPF1 and total UPF1. **g** Effect of NPD15008 on NMD activity. Levels of SNHG1 mRNA, an endogenous NMD substrate, were analyzed following NPD15008 treatment. Data are from three independent experiments and are presented as the mean ± standard error

Next, we evaluated the ability of these compounds to inhibit NMD using a cell-based luciferase reporter system in which a single reporter cassette is integrated at an identical genomic locus (Supplementary Fig. 1d). Through this analysis, we identified one compound, NPD15008, derived from the Natural Products Depository (NPDepo), as a potent SMG1 inhibitor (Fig. 1a and Supplementary Fig. 1c, e–g). NPD15008 exhibited an IC_50_ of 128 nM or 1263 nM at ATP concentrations of 1 μM and 10 μM, respectively, indicating that it functions as an ATP-competitive inhibitor (Fig. 1b, Supplementary Fig. 1e–g, Supplementary Table 1). Using microscale thermophoresis, we further determined that NPD15008 binds directly to SMG1 with a dissociation constant (*K*_D_) of 29 nM (Fig. 1d).

While these studies were ongoing, compound 11j was reported as an SMG1/mTOR inhibitor.^33^ We therefore synthesized compound 11j and analyzed it in our assay system. Compound 11j exhibited IC_50_ values of 160 nM for SMG1, 8.7 μM for mTOR, and 43.9 μM for DNA-PK in the presence of 10 μM ATP. In addition, we found that the commercially available polo-like kinase inhibitor GW-843682X also inhibited SMG1, with an IC_50_ of 3.2 μM (Fig. 1c and Supplementary Table 1). Binding analyses revealed that ATP, compound 11j, and GW-843682X bound to SMG1 with calculated *K*_D_ values of 273 μM, 9.48 nM, and 205 nM, respectively (Supplementary Fig. 2a–c).

To assess kinase selectivity at broader scale, NPD15008, compound 11j, and GW-843682X were subjected to KINOMEscan profiling^34^ at a concentration of 10 μM against panels of 97 or 468 distinct kinases. NPD15008 exhibited specificity comparable to that of the other two synthetic reference compounds (Supplementary Fig. 2d–g and Supplementary Table 2). Given its potency and favorable selectivity profile, we selected NPD15008 as the most suitable compound for investigating the acute cellular effects of SMG1 inhibition.

We next examined whether NPD15008 inhibits SMG1 kinase activity in cells by monitoring autophosphorylation of SMG1 at T3550, a modification within the C-terminal region that correlates with SMG1 kinase activity.^35^ To detect this phosphorylation, we generated a monoclonal antibody, SpMab-7, that specifically recognizes SMG1 phosphorylated at T3550 (P-SMG1). SpMab-7 detected endogenous phosphorylation, which was enhanced by SMG1 overexpression and markedly decreased upon treatment with NPD15008 (Fig. 1e). Consistently, the signal was also reduced upon expression of kinase-inactive SMG1 mutant (SMG1-DA; aspartic acid 2335 replaced with alanine) (Supplementary Fig. 5q). These results confirm that SpMab-7 specifically detects SMG1 autophosphorylation and that NPD15008 effectively suppresses SMG1 kinase activity in cells. Consistent with SMG1 autophosphorylation, phosphorylation of UPF1, a canonical SMG1 substrate, was increased upon SMG1 overexpression and suppressed by NPD15008 treatment (Fig. 1e). As previously reported,^35^ SMG1-mediated phosphorylation of UPF1 is a key step in activating NMD. Dose-dependent treatment with NPD15008 reduced UPF1 phosphorylation in HeLa Tet-Off cells, with an IC_50_ of 568 nM (Fig. 1f). Accordingly, NPD15008 induced accumulation of SNHG1 mRNA, an endogenous NMD target, with an IC_50_ of 1283 nM (Fig. 1g). Together, these data demonstrate that NPD15008 is a potent and selective SMG1 inhibitor that suppresses both SMG1 kinase activity and NMD in cellular contexts.

### SMG1 inhibition induces ferroptosis-associated oxidative stress

**Fig. 2:**
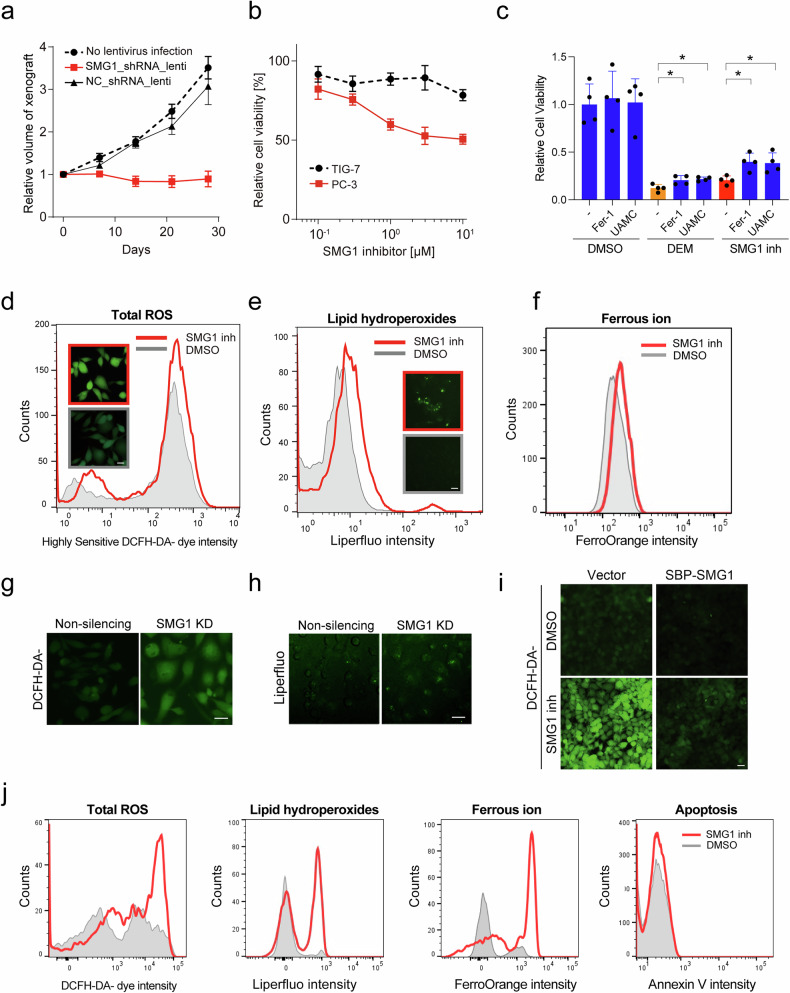
SMG1 inhibition suppresses tumor growth and induces ferroptotic cell death through oxidative stress accumulation. **a** Stable SMG1 knockdown suppresses PC-3 xenograft tumor growth in nude mice. Nude mice (nu/nu) were inoculated with PC-3 cells and treated with intratumoral injections of lentivirus expressing SMG1-targeting shRNA (SMG1_shRNA_lenti) (squares), Rluc non-silencing control shRNA (NC_shRNA_lenti) (triangles), or no lentivirus (circles), beginning one week after tumor cell inoculation. Tumor volumes are shown as the mean ± standard error (*n* = 6–10 tumors per group). **b** Cell viability of PC-3 prostate cancer cells and normal human fibroblasts (TIG-7) treated with NPD15008 for 24 h. Viability was assessed using the alamarBlue assay. Data were obtained from pentaplicate samples across three biologically independent experiments and are presented as the mean ± standard error. **c** Effects of ferroptosis inhibitors on NPD15008- or DEM-induced cell death. PC-3 cells were treated with 5 μM ferrostatin-1 or 0.01 μM UAMC-3203 in combination with 6 μM NPD15008 or 200 μM DEM for 48 h. Cell viability was determined by counting viable cells using ImageJ2 (Fiji) software. Data were obtained from pentaplicate samples across four biologically independent experiments and are presented as the mean ± standard error. Statistical analysis was performed using one-way ANOVA followed by Sidak’s multiple-comparison test. *P < 0.05. **d**–**f** Flow cytometric analysis of PC-3 cells treated with or without 6 μM NPD15008 for 24 h. Representative results from three biologically independent experiments are shown. **d** Intracellular total reactive oxygen species (ROS) levels measured using the DCFH-DA probe. **e** Lipid hydroperoxide levels measured using the Liperfluo probe. **f** Intracellular ferrous iron levels measured using the FerroOrange probe. **d**, **e** Insets show representative fluorescence microscopy images. Scale bar, 20 μm. **g**, **h** PC-3 cells transfected with SMG1-targeting or control siRNA for 48 h. **g** Intracellular total ROS levels measured using the photo-oxidation–resistant DCFH-DA probe. **h** Lipid hydroperoxide levels measured using the Liperfluo probe. Knockdown efficiencies of SMG1 in (**g**, **h**) were confirmed by immunoblotting (see Supplementary Fig. 7o). **i** Intracellular total ROS levels in HEK293T cells overexpressing SMG1, measured using the photo-oxidation resistant DCFH-DA probe. Forty-eight hours after transfection, cells were treated with or without NPD15008. **j** Flow cytometric analysis of intracellular total ROS, lipid hydroperoxide, ferrous iron, and annexin V levels in mouse liver cells. Mice were treated with NPD15008 or DMSO control for 8 h

We previously reported that SMG1 knockdown reduces the viability of multiple cancer cell lines through an undefined mechanism, whereas its impact is less pronounced in normal human fibroblasts.^36,37^ To evaluate the contribution of SMG1 to tumor growth in vivo, we examined the effect of stable SMG1 knockdown in PC-3 prostate cancer xenografts. PC-3 cells were implanted into nude mice, and once the xenografts reached an average volume of ~65 mm^3^, tumors were infected in situ with lentiviruses expressing shRNA targeting either SMG1 or Renilla luciferase (Rluc) as a non-silencing control (NC). A marked reduction in xenograft growth was observed in the SMG1 shRNA–treated group, whereas tumors infected with NC control shRNA continued to grow, indicating that SMG1 is required for intrinsic cancer cell growth (Fig. 2a). We next evaluated the effect of pharmacological SMG1 inhibition on tumor progression using NPD15008. Treatment with NPD15008 showed a modest trend toward reduced growth of PC-3 xenografts (Supplementary Fig. 3a). However, robust and reproducible anti-tumor effects were not consistently observed in vivo, likely reflecting the limited solubility and bioavailability of NPD15008, which restricted sustained systemic exposure required for long-term therapeutic evaluation. Nevertheless, NPD15008 reproducibly induced acute molecular responses within defined short time frames and was therefore suitable for interrogating SMG1-dependent signaling events in cultured cells and short-term in vivo settings.

To elucidate the mechanism underlying reduction in cell viability upon SMG1 inhibition, we examined the effects of NPD15008 in PC-3 prostate cancer cells and TIG-7 normal human fibroblasts. NPD15008 markedly reduced cell viability in PC-3 cells, whereas its effect was substantially weaker in TIG-7 cells (Fig. 2b), suggesting that SMG1 inhibition preferentially engages cell viability–suppressing pathways in cancer cells. We therefore investigated which regulated cell death pathways contribute to the loss of viability. Neither the pan-caspase inhibitor Z-VAD-FMK nor the necroptosis inhibitor necrostatin-1 restored cell viability (Supplementary Fig. 3b). In contrast, two structurally distinct ferroptosis inhibitors, ferrostatin-1 (Fer-1) and UAMC-3203 (UAMC) partially rescued cell viability (Fig. 2c). Consistent with these pharmacological findings, SMG1 inhibition induced accumulation of 4-Hydroxynonenal (4-HNE)-modified proteins, a biochemical marker of lipid peroxidation, whereas the proteasome inhibitor bortezomib did not (Supplementary Fig. 3c). Moreover, canonical markers of apoptosis or necroptosis—including cleaved caspase-3 and phospho-MLKL—were not detected under these conditions (Supplementary Fig. 3c). Together, these findings suggest that SMG1 inhibition is associated with lipid peroxidation in the absence of dominant apoptotic or necroptotic signaling. The partial rescue by ferroptosis inhibitors prompted us to compare the effects of SMG1 inhibition with those of established ferroptosis inducers. Ferroptosis induced by ML162, a GPX4 inhibitor,^38^ was largely suppressed by ferroptosis inhibitors (Supplementary Fig. 3b). In contrast, diethyl maleate (DEM), a glutathione-depleting agent^39^ (Supplementary Fig. 3d), phenocopied key aspects of SMG1 inhibition (Fig. 2c and Supplementary Fig. 3c), suggesting that SMG1 inhibition induces oxidative stress comparable to glutathione depletion. To further substantiate this possibility, we assessed intracellular ROS accumulation, lipid hydroperoxides, and ferrous iron using specific fluorescent probes: 2’,7’- dichlorodihydrofluorescein diacetate (DCFH-DA), Liperfluo, and FerroOrange, respectively. Notably, Liperfluo selectively detects lipid hydroperoxides, a hallmark biochemical feature of ferroptosis. Flow cytometry and fluorescence imaging analyses revealed that NPD15008 increased intracellular total ROS (Fig. 2d and Supplementary Fig. 3e), lipid hydroperoxides (Fig. 2e and Supplementary Fig. 3f), and ferrous iron levels (Fig. 2f), without apoptosis marker annexin V positivity (Supplementary Fig. 3g). Similarly, DEM treatment induced accumulation of total ROS and lipid hydroperoxide (Supplementary Fig. 3h, i). To define the role of SMG1 in regulating oxidative stress, we next employed genetic approaches. SMG1 knockdown increased intracellular total ROS and lipid hydroperoxide levels (Fig. 2g, h and Supplementary Fig. 7o), whereas SMG1 overexpression suppressed ROS accumulation in a kinase activity–dependent manner, as this effect was abolished by NPD15008 (Fig. 2i). Finally, to extend these findings in vivo, we assessed ferroptosis-associated biochemical markers in mouse liver following short-term NPD15008 administration. Flow cytometric analysis revealed increased total ROS, lipid hydroperoxides, and ferrous iron, without annexin V positivity (Fig. 2j). While these in vivo data do not establish ferroptosis as the exclusive mode of cell death, they are consistent with acute ferroptosis-associated molecular changes induced by SMG1 inhibition.

Collectively, biochemical (4-HNE, total ROS, lipid hydroperoxides, and ferrous iron accumulation), pharmacological (selective rescue by ferroptosis inhibitors), genetic (SMG1 knockdown and gain-of-function), and in vivo analyses converge to support a model in which SMG1 constrains oxidative stress and limits susceptibility to ferroptosis, without evidence for dominant contributions from apoptosis or necroptosis under the conditions examined.

### SMG1–NRF2 signaling is preferentially engaged under mild oxidative stress

Given that SMG1 activity promotes cell survival by limiting intracellular ROS accumulation, we hypothesized that SMG1 selectively regulates redox-responsive signaling pathways depending on the intensity of oxidative stress.

**Fig. 3:**
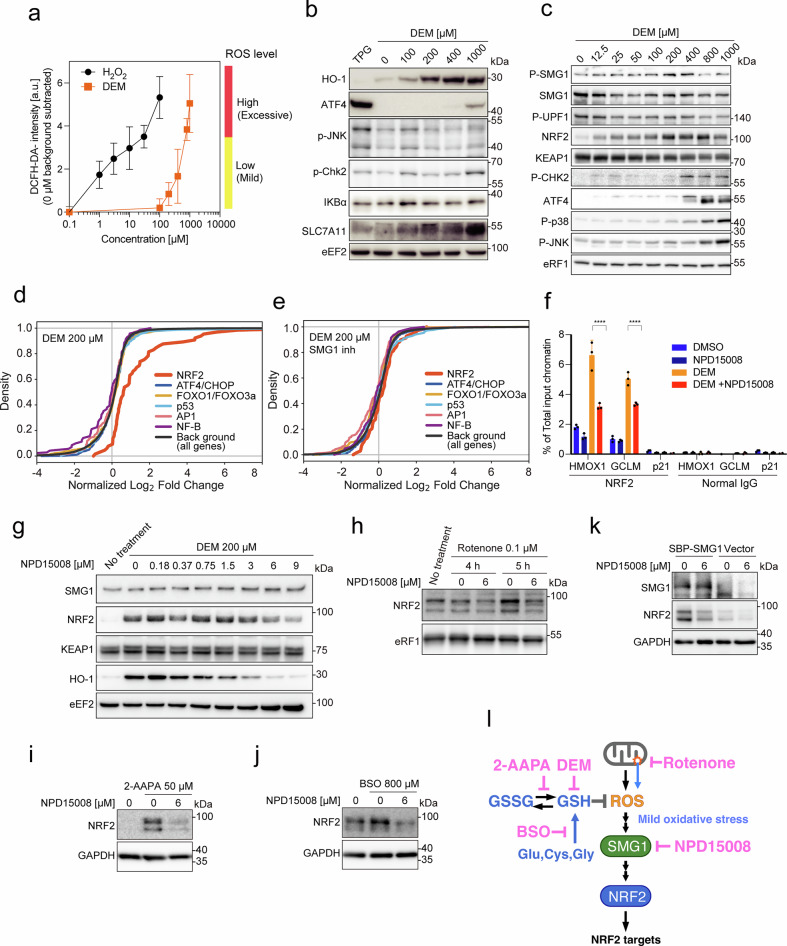
Stress–intensity–dependent SMG1 kinase activation selectively drives NRF2 signaling. **a** Intracellular total ROS levels were estimated using DCFH-DA–based fluorescence as a semi-quantitative measure of cellular ROS accumulation. PC-3 cells were treated with increasing concentrations of DEM (0–1000 μM) for 4 h or exposed to exogenous hydrogen peroxide (0–100 μM) prior to staining for calibration purposes. DCFH-DA fluorescence signals were acquired by fluorescence microscopy and quantified using ImageJ2 (Fiji) software. Signals obtained at 0 μM were subtracted as background and plotted as indicated. These measurements were used to operationally define mild and excessive oxidative stress conditions based on intracellular ROS accumulation and activation of anti-survival stress pathways. **b** PC-3 cells were treated with serial dilutions of DEM for 4 h. Total cell extracts were subjected to immunoblotting with the indicated antibodies to assess pro-survival antioxidant proteins (HO-1, SLC7A11, and IκBα) and anti-survival stress markers (ATF4, phospho-JNK, and phospho-CHK2). eEF2 served as a loading control. **c** HepG2 cells were treated with serial dilutions of DEM for 3 h. Total cell extracts were subjected to immunoblotting with the indicated antibodies to assess NRF2 and KEAP1 accumulation, activation of anti-survival stress markers (ATF4, phospho-JNK, phospho-p38, and phospho-CHK2); and SMG1 kinase autophosphorylation, which was enhanced under mild oxidative stress and suppressed under excessive oxidative stress. eRF1 served as a loading control. **d**, **e** Cumulative distributions of log₂ fold changes in de novo-transcribed mRNAs for selected transcription factor target gene sets relative to all expressed genes (background). PC-3 cells were treated with 200 μM DEM alone (**d**) or in combination with 9 μM NPD15008 (**e**) for 3 h. Statistical significance was assessed using adjusted Welch’s t-tests. NRF2 target genes exhibited a significant rightward shift following DEM treatment, which was attenuated by 9 µM NPD15008, whereas target genes of other stress-responsive transcription factors showed minimal or no induction. Adjusted Welch’s t-test P values: NRF2 transcriptional targets (*P* = 7.26 × 10^–6^ in (**d**) and *P* = 0.12 in (**e**)), ATF4 and C/EBP homologous protein (ATF4/CHOP) transcriptional targets (*P* = 0.25 in (**d**) and *P* = 1.0 in (**e**)), Forkhead box O1 and 3a (FOXO1/FOXO3a) transcriptional targets (*P* = 1.00 in (**d**) and *P* = 0.06 in (**e**)), p53 transcriptional targets (*P* = 1.00 in both (**d**) and (**e**)), AP-1 transcriptional targets (*P* = 0.74 in (**d**) and *P* = 0.001 in (**e**)), and NF-κB transcriptional targets (*P* = 0.18 in (**d**) and *P* = 0.85 in (**e**)). **f** Effect of SMG1 inhibition on NRF2 chromatin occupancy. NRF2 binding at the HMOX1 (HO-1) enhancer and GCLM promoter regions, as well as at a p21 upstream negative control region, was assessed by ChIP-qPCR using chromatin isolated from PC-3 cells treated with 200 µM DEM in the presence or absence of NPD15008 for 4 h. Chromatin was immunoprecipitated with an anti-NRF2 antibody, and enrichment was quantified by qPCR. **g** SMG1 inhibition suppresses DEM-induced NRF2 accumulation. PC-3 cells were treated with 200 μM DEM together with serial dilutions of NPD15008 for 4 h. Whole-cell lysates were analyzed by immunoblotting with the indicated antibodies. KEAP1 protein levels are shown. eEF2 served as a loading control. SMG1 inhibition attenuates NRF2 accumulation induced by diverse oxidative stressors. PC-3 cells were treated with 0.1 µM rotenone (**h**), 50 µM 2-AAPA (**i**), or 800 µM BSO (**j**) in the presence or absence of NPD15008 for 4–5 h as indicated. Total cell extracts were analyzed by immunoblotting with the indicated antibodies. **k** SMG1 kinase activity is sufficient to promote NRF2 accumulation. Experiments were performed together with those shown in Fig. 1e. Whole-cell lysates were analyzed by immunoblotting with the indicated antibodies. **l** Model illustrating stress–intensity–dependent engagement of SMG1-NRF2 signaling. Under mild oxidative stress, limited intracellular peroxide accumulation enhances SMG1 kinase activity, promoting NRF2 stabilization and transcription of pro-survival and anti-ferroptotic genes. Unless otherwise indicated, values represent the mean ± SD. Statistical analysis was performed using one-way ANOVA with Tukey’s multiple-comparison test. **P < 0.01, ***P < 0.001, ****P < 0.0001

In this study, oxidative stress intensity was operationally defined based on intracellular total ROS accumulation and activation of canonical anti-survival stress pathways (see Materials and Methods). To experimentally distinguish between levels of oxidative stress, PC-3 cells were exposed to increasing concentrations of diethyl maleate (DEM) for 4 h, and intracellular total ROS accumulation was assessed using DCFH-DA–based fluorescence analysis. Low concentrations of DEM induced a limited increase in intracellular total ROS, which we defined as mild oxidative stress, whereas substantially higher concentrations were required to reach ROS levels associated with activation of ATM–CHK2 and JNK signaling pathways, which we defined as excessive oxidative stress (Fig. 3a and Supplementary Fig. 4a).

Having established these operational definitions, we next examined the expression of pro-survival antioxidant proteins (HO-1, SLC7A11, and IκBα) and anti-survival stress markers (ATF4, phospho-JNK, and phospho-CHK2) under these conditions in PC-3 cells.^16,40–42^ Under conditions corresponding to mild oxidative stress, HO-1 expression was induced, whereas activation of the anti-survival transcription factor ATF4 was observed only under excessive oxidative stress (Fig. 3b and Supplementary Fig. 4b). SLC7A11 expression showed modest induction under mild oxidative stress and a pronounced increase only under excessive oxidative stress, while phosphorylation of CHK2 and JNK was detected exclusively under excessive oxidative stress (Fig. 3b and Supplementary Fig. 4c). In contrast, IκBα expression remained largely unchanged across these conditions (Fig. 3b and Supplementary Fig. 4b). By comparison, treatment with thapsigargin, an inducer of endoplasmic reticulum stress, robustly activated ATF4 and phospho-JNK with minimal effects on HO-1 or phospho-CHK2 (Fig. 3b), indicating that DEM elicits a distinct, dose-dependent redox signaling program than a generalized stress response. Consistent with the DEM results, treatment with the glutathione reductase inhibitor 2-AAPA induced NRF2 accumulation at lower concentrations, whereas ATF4 and p38 activation were observed only at higher concentrations (Supplementary Fig. 4c, d). The glutamate-cysteine ligase inhibitor L-buthionine-(S,R)-sulfoximine (BSO) dose-dependently induced NRF2 accumulation, albeit less efficiently than DEM or 2-AAPA, and did not induce ATF4 and stress kinase pathways (Supplementary Fig. 4e). PC-3 cells harbor multiple mutations and loss of heterozygosity in genes involved in oxidative stress responses, including *TP53*.^43,44^ To exclude the possibility that this stress–intensity–dependent signaling behavior is cell line–specific, we performed parallel experiments in HepG2 hepatocellular carcinoma cells, which lack such mutations.^45^ In HepG2 cells, DEM treatment induced NRF2 accumulation under conditions corresponding to mild oxidative stress, whereas higher DEM concentrations corresponding to excessive oxidative stress led to a reduction in NRF2 levels concomitant with induction of ATF4, phospho-JNK, phospho-p38, and phospho-CHK2 (Fig. 3c). Under these excessive stress conditions, KEAP1 protein levels were modestly decreased (Fig. 3c). This observation is consistent with a recent report describing CHK2-dependent modulation of KEAP1 under high oxidative stress conditions^46^ although the precise mechanism was not examined here. Similarly, 2-AAPA induced NRF2 accumulation at lower concentrations, while higher concentrations preferentially activated ATF4 and stress kinase pathways (Supplementary Fig. 4d). BSO induced NRF2 accumulation at higher concentrations and did not induce ATF4 and stress kinase pathways (Supplementary Fig. 4f). Notably, SMG1 kinase autophosphorylation was enhanced under mild oxidative stress but suppressed under excessive oxidative stress (Fig. 3c), supporting a model in which SMG1 activity is selectively engaged under conditions of limited oxidative stress. However, PKC autophosphorylation was not enhanced in both mild and excessive oxidative stress caused by DEM treatment (Supplementary Fig. 4g).

Collectively, NRF2 preferentially accumulated under mild oxidative stress, whereas ATF4, phospho-JNK/phospho-p38, and phospho-CHK2 accumulated under excessive oxidative stress in both PC-3 and HepG2 cells. These results indicate that SMG1–NRF2 signaling is preferentially engaged under conditions of mild oxidative stress characterized by modest intracellular total ROS accumulation, while excessive oxidative stress overrides this pro-survival pathway and activates anti-survival stress responses.

Consistent with these observations, global de novo transcriptome analysis revealed that among major oxidative stress–responsive transcription factors—including NRF2, ATF4/CHOP, FOXO1/FOXO3a, p53, AP-1, and NF-κB,^1,47,48^ only NRF2-dependent gene expression was significantly induced under mild oxidative stress (Fig. 3d). Importantly, this NRF2-driven transcriptional program was markedly attenuated by pharmacological SMG1 inhibition with NPD15008 (Fig. 3e). Induction of representative NRF2 target genes, including HO-1 and GCLM, was confirmed by both de novo RNA sequencing and RT–qPCR (Supplementary Fig. 5a, b). Notably, mild oxidative stress did not induce accumulation of NMD target transcripts such as *ATF4* or *SNHG1*, in contrast to the pronounced accumulation observed following SMG1 inhibition (Supplementary Fig. 5c, d), indicating that NRF2 activation under these conditions occurs independently of global NMD suppression. Consistent with transcriptional activation, chromatin immunoprecipitation followed by qPCR revealed enhanced NRF2 occupancy at the *HMOX1* (HO-1) enhancer and *GCLM* promoter regions under mild oxidative stress, and this enrichment was significantly reduced by NPD15008 treatment (Fig. 3f). Global de novo transcriptome analysis further confirmed the induction of anti-ferroptotic gene expression, but not pro- or anti-apoptotic or necroptotic gene expression, in response to mild oxidative stress, and this transcriptional program was suppressed by SMG1 inhibition (Supplementary Fig. 5e–h).

At the protein level, SMG1 inhibition by knockdown (Supplementary Fig. 5i), NPD15008 (Fig. 3g), or compound 11j suppressed DEM-induced accumulation of NRF2 and HO-1 in a dose-dependent manner (Supplementary Fig. 5j). Neither DEM treatment nor SMG1 inhibition altered KEAP1 protein levels, indicating that SMG1-dependent NRF2 accumulation under these conditions is not accompanied by detectable KEAP1 degradation (Fig. 3g). To determine whether this requirement for SMG1 extends beyond DEM-induced glutathione depletion, we examined additional oxidative stress stimuli. SMG1 inhibition attenuated NRF2 accumulation induced by BSO and 2-AAPA, as well as by low-dose rotenone, sodium arsenite, and tert-butylhydroquinone (Fig. 3h–j and Supplementary Fig. 5k–p). These findings indicate that SMG1 activity broadly supports NRF2 activation in response to diverse sources of oxidative stress.

Finally, overexpression of wild-type SMG1 in HEK293T cells was sufficient to increase NRF2 protein levels, whereas this effect was abolished by pharmacological SMG1 inhibition and was not recapitulated by a kinase-inactive SMG1 mutant (Fig. 3k and Supplementary Fig. 5q). Together, these results indicate that SMG1 kinase activity is both necessary and sufficient to promote NRF2 accumulation under conditions of limited oxidative stress.

Collectively, these data support a model in which SMG1 kinase activity is selectively engaged under mild oxidative stress, thereby promoting NRF2-dependent pro-survival signaling, whereas excessive oxidative stress suppresses this pathway and redirects signaling toward anti-survival stress responses (Fig. 3l).

### SMG1 directly phosphorylates NRF2 in vitro and in intact cells in response to mild oxidative stress

**Fig. 4:**
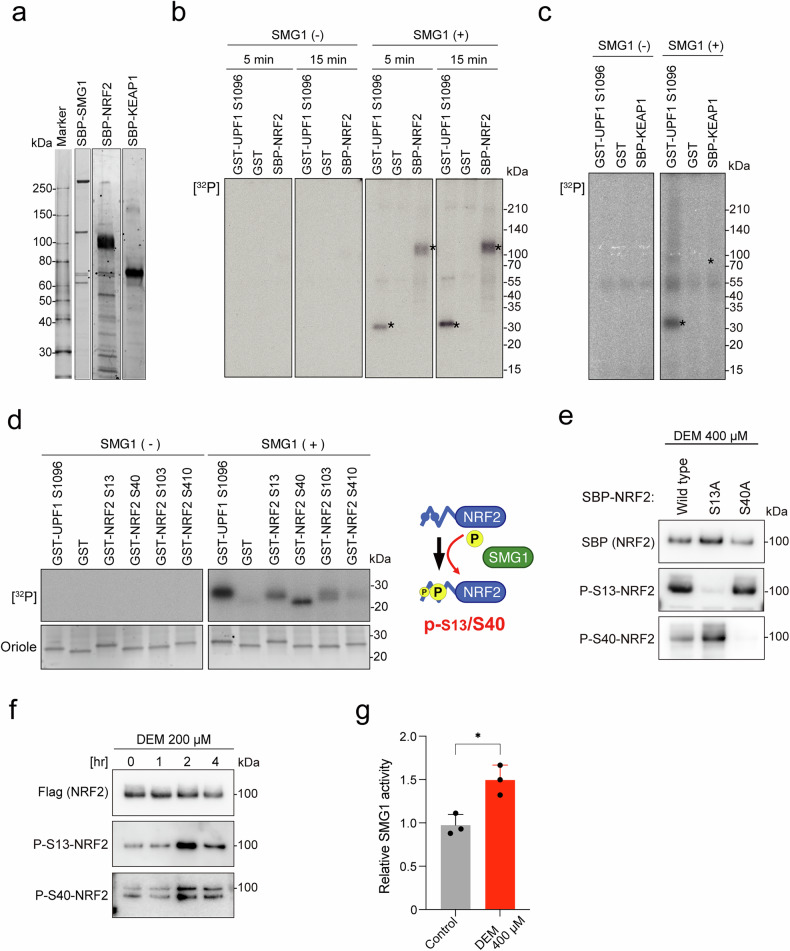
SMG1 directly and selectively phosphorylates NRF2 at S13 and S40 in vitro and in cells. **a** Oriole-stained SDS-PAGE gels of the purified proteins used in (**b**–**d**). In vitro kinase assay of SMG1. **b** In vitro kinase assays using purified SMG1C and purified NRF2 shown in (**a**). SMG1 directly phosphorylates NRF2. Asterisks indicate phosphorylated NRF2 or UPF1 in autoradiograms. **c** SMG1 does not phosphorylate KEAP1 in vitro. In vitro kinase assays using purified SBP-KEAP1 shown in (**a**). Asterisks indicate phosphorylated UPF1 or the position of KEAP1 in the corresponding lanes. **d** SMG1 phosphorylates GST-fused 14-mer SQ-containing peptides derived from NRF2. In vitro kinase assays were performed using GST-fused peptides. Numbers indicate the serine residues of NRF2. **e** Analysis of phosphorylation of S13 and S40 residues of NRF2 in response to DEM. SBP-NRF2 wild-type, S13A or S40A purified from 293 T cells treated with 400 μM DEM for 2 h were probed with the indicated antibodies. **f** In-cell phosphorylation analysis of S13 and S40 residues of NRF2 in response to DEM. Flag-tagged NRF2 wild-type purified from 293 T cells treated with 200 μM DEM for the indicated time course were probed with the indicated antibodies. Total levels of Flag-NRF2 were not altered by DEM under these conditions. **g** Quantification of DEM-induced SMG1 activation. PC-3 cells were treated with 400 μM DEM for 2 h. Endogenous SMG1 was immunopurified using an anti-SMG1 antibody and protein-G-Dynabeads. Kinase activities of the SMG1 immuno-complex were determined by the extent of phosphorylation of the UPF1 peptide substrate. Phosphorylation of the substrate was detected with the liquid scintillation counter. Details are described in “Materials and Methods”. Data are from three independent experiments and are shown as the mean ± standard error. * *P* < 0.01, *t* test (*n* = 3). Unless otherwise indicated, values represent mean ± SD. Statistical analysis was performed using one-way ANOVA with Tukey’s multiple-comparison test. **P < 0.01, ***P < 0.001, ****P < 0.0001

To elucidate the direct effect of SMG1 kinase activity on NRF2, we first examined whether SMG1 could directly phosphorylate NRF2 and/or KEAP1 in vitro. We purified full-length SBP-tagged SMG1–SMG8–SMG9 complex (SMG1C), the active ternary kinase complex of SMG1,^49^ as well as recombinant NRF2 and KEAP1 (Fig. 4a). In vitro kinase assays demonstrated that SMG1C directly phosphorylated full-length NRF2, but not KEAP1 (Fig. 4b, c). Since SMG1 exhibits a strict substrate preference for serine (S) or threonine (T) residues followed by a glutamine (Q) residue (the S/TQ motif),^19,35^ we analyzed all four S/TQ motifs present in NRF2. To this end, we generated a series of GST-fusion peptides containing NRF2 sequences encompassing each S/TQ with six flanking amino acids on either side. SMG1 efficiently phosphorylated a control substrate containing the SQ motif in UPF1-S1096 and NRF2-S40 to a similar extent, whereas NRF2 phosphorylation at S13 was weaker, and that at S103 and S410 was minimal (Fig. 4d).

To assess phosphorylation of NRF2 at S13 and S40 in intact cells, we generated phospho-specific polyclonal antibodies recognizing phosphorylated S13 (P-S13-NRF2). Together with a commercially available phospho-specific antibody against phosphorylated S40 (P-S40-NRF2), phosphorylation at both residues was detected in overexpressed SBP-tagged wild-type NRF2, but not in the corresponding serine-to-alanine mutants, in HEK293T cells subjected to DEM treatment (Fig. 4e), validating antibody specificity. These results demonstrate that both residues can be phosphorylated in cells under mild oxidative conditions. Consistently, phosphorylation at S13 and S40 was enhanced in Flag-tagged NRF2 following DEM treatment (Fig. 4f). Consistent with this, the kinase activity of immunoprecipitated endogenous SMG1 was enhanced in response to mild oxidative stress in PC-3 cells (Fig. 4g). Collectively, these findings, together with the in vitro kinase assays, support a model in which SMG1 directly phosphorylates NRF2 in response to mild oxidative stress. S40 represents a physiologically detectable phosphorylation site in intact cells, whereas S13 may function as an additional regulatory site.

### SMG1-mediated phosphorylation of NRF2 is required for NRF2 accumulation

**Fig. 5:**
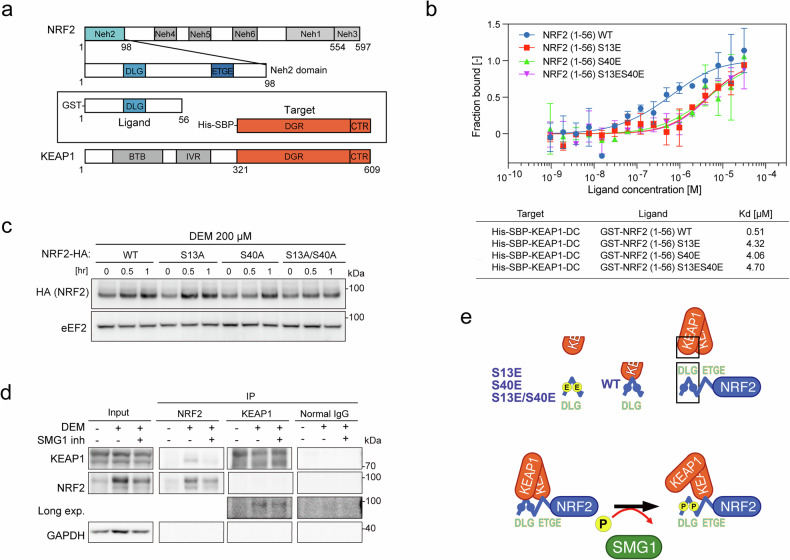
SMG1-mediated phosphorylation of NRF2 at S13 and S40 impairs KEAP1 interaction and drives NRF2 accumulation. **a** Schematic representation of NRF2 and KEAP1 domains used for binding analysis. **b** Microscale thermophoresis analysis of the interaction between KEAP1 and NRF2. Fluorescently labeled KEAP1 (residues 321–609) was titrated with NRF2 (residues 1–56) wild-type (blue), S13E (red), S40E (green), or the S13E/S40E double mutant (purple). Dissociation constants (*K*_*D*_) are presented as the mean ± standard error of two independent experiments. **c** Effect of S13 and/or S40 substitution on DEM-induced NRF2 protein accumulation. PC-3 cells transiently expressing NRF2-HA wild-type, S13A, S40A, or S13A/S40A were treated with 200 μM DEM for the indicated times. Whole-cell lysates were analyzed by immunoblotting with the indicated antibodies. eEF2 served as a loading control. The blot shown is representative of three independent experiments. **d** Analysis of the NRF2–KEAP1 interaction in response to DEM and SMG1 inhibition. HepG2 cells were treated with 25 μM DEM in the presence or absence of NPD15008. Cell lysates were subjected to immunoprecipitation using antibodies against NRF2 or KEAP1, followed by immunoblotting with the indicated antibodies. **e** Schematic representation of the phospho-mimetic NRF2 DLG motif with attenuated affinity for KEAP1 (upper). Model depicting SMG1-mediated phosphorylation of S13 and/or S40 attenuating DLG motif–KEAP1 binding while the ETGE motif–KEAP1 interaction is retained, thereby maintaining overall NRF2–KEAP1 association (bottom)

We next examined the functional consequences of SMG1-mediated phosphorylation of NRF2 at S13 and S40. Both residues are located in close proximity to the DLG motif of NRF2, one of the two KEAP1-binding motifs essential for NRF2 ubiquitination (Fig. 5a).^11,12^ We therefore analyzed the interaction between the KEAP1 Kelch domain (residues 321–609) and a NRF2 fragment encompassing the DLG motif (residues 1–56), either in its wild-type form or containing phospho-mimetic substitutions (Fig. 5a and Supplementary Fig. 6a–c). The NRF2 (1–56) fragment bound directly to KEAP1 (321–609) with a dissociation constant (*K*_D_) of 0.5 μM. In contrast, phospho-mimetic mutants (S13E, S40E or S13E/S40E double mutant) showed markedly reduced affinity for KEAP1, with *K*_D_ values of 4.3 μM, 4.0 μM and 4.7 μM, respectively (Fig. 5b). These results indicate that phosphorylation of NRF2 at either S13 or S40 weakens the DLG-mediated interaction between NRF2 and KEAP1 (Fig.5e, upper).

We next assessed NRF2 accumulation using phosphorylation-deficient mutants under mild oxidative stress conditions. HA-tagged wild-type NRF2 or serine-to-alanine mutants (S13A, S40A, or S13A/S40A) were transiently expressed in PC-3 cells, followed by DEM treatment. NRF2 accumulation induced by DEM was comparable between wild-type NRF2 and the single mutants, whereas the S13A/S40A double mutant exhibited reduced accumulation (Fig. 5c). These results indicate that phosphorylation at either S13 or S40 is sufficient to support NRF2 accumulation, consistent with the reduced KEAP1-binding affinity observed for the corresponding phospho-mimetic mutants in the context of DLG motif.

NRF2 also contains the ETGE motif that mediates a constitutive high-affinity anchoring interaction with KEAP1.^11,12^ Consistent with this, the overall NRF2–KEAP1 association remained largely unchanged following mild oxidative stress, with or without SMG1 inhibition, as assessed by immunoprecipitation of endogenous NRF2 or KEAP1 in HepG2 cells (Fig. 5d).

Together, these results indicate that SMG1-mediated phosphorylation of NRF2 promotes NRF2 accumulation by attenuating DLG-dependent interaction with KEAP1 without disrupting ETGE-mediated anchoring (Fig. 5e, bottom).

### DNA-PK functions redundantly with SMG1 to activate NRF2 under mild oxidative stress

**Fig. 6:**
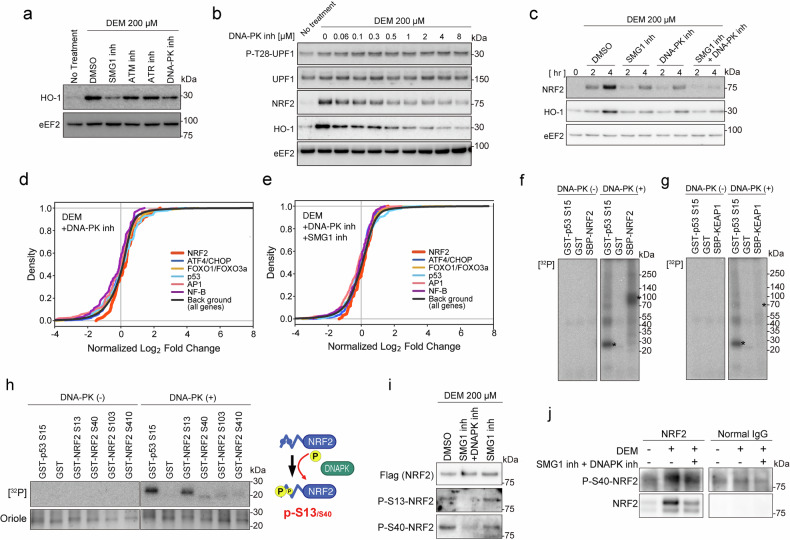
DNA-PK cooperates with SMG1 to phosphorylate NRF2 and activate antioxidant transcription. **a** Contribution of PIKK family kinases to DEM-induced HO-1 accumulation. PC-3 cells were treated with 200 μM DEM together with the indicated PIKK inhibitors [6 μM NPD15008 (SMG1 inhibitor), 10 μM KU-55933 (ATM inhibitor), 10 μM VE-821 (ATR inhibitor), 2 μM NU7441 (DNA-PK inhibitor)] for 4 h. Whole-cell lysates were analyzed by immunoblotting with the indicated antibodies. eEF2 served as a loading control. Representative blots from four independent experiments are shown. **b** Dose-dependent suppression of DEM-induced NRF2 and HO-1 accumulation by NU7441. PC-3 cells were treated with 200 μM DEM together with increasing concentrations of NU7441 for 4 h. Whole-cell lysates were analyzed by immunoblotting. UPF1 phosphorylation is shown to assess NMD activity. eEF2 served as a loading control. Representative results from three independent experiments are shown. **c** Additive suppression of NRF2 and HO-1 accumulation by combined SMG1 and DNA-PK inhibition. PC-3 cells were treated with 200 μM DEM in the presence of 6 μM NPD15008 and/or 2 μM NU7441 for the indicated times. Whole-cell lysates were analyzed by immunoblotting. eEF2 served as a loading control. **d**, **e** Cumulative distributions of log₂ fold changes in de novo–transcribed mRNAs for selected transcription factor target gene sets relative to all expressed genes (background). PC-3 cells were treated with 200 μM DEM plus 4 μM NU7441 (**d**) or 200 μM DEM plus 4 μM NU7441 and 9 μM NPD15008 (**e**) for 3 h. Statistical significance was assessed using adjusted Welch’s t-tests. NRF2 target genes exhibited significant induction upon DEM treatment that was suppressed by DNA-PK inhibition, with modest additive suppression upon co-treatment with NPD15008. Adjusted Welch’s *t* test *P* values: NRF2 transcriptional targets (*P* = 0.07 in (**d**) and *P* = 1.00 in (**e**)), ATF4/CHOP transcriptional targets (*P* = 1.00 in (**d**) and *P* = 0.91 in (**e**)), FOXO1/FOXO3a transcriptional targets (*P* = 1.00 in (**d**) and *P* = 0.68 in (**e**)), p53 transcriptional targets (*P* = 1.00 in both (**d** and **e**)), AP-1 transcriptional targets (*P* = 0.09 in (**d**) and *P* = 0.001 in (**e**)), and NF-κB transcriptional targets (*P* = 0.02 in (**d**) and *P* = 0.29 in (**e**)). Experiments were performed together with those shown in Fig. 3 d, 3e; therefore, identical DMSO, DEM, and DEM + SMG1 inhibitor datasets were used for comparative analysis. RNA-seq data are provided in Supplementary Tables 3 and 4. **f** DNA-PK directly phosphorylates NRF2 in vitro. Purified DNA-PK and recombinant NRF2 were subjected to in vitro kinase assays, and phosphorylation was detected by autoradiography. Asterisks indicate phosphorylated NRF2 and p53 (positive control). **g** DNA-PK does not phosphorylate KEAP1 in vitro. In vitro kinase assays were performed using purified SBP-KEAP1 as the substrate. The asterisk indicates phosphorylated p53 (positive control). **h** DNA-PK preferentially phosphorylates NRF2-S13. In vitro kinase assays were performed using GST-fused peptides encompassing individual SQ motifs of NRF2. Phosphorylation efficiency was compared with that of the canonical substrate p53-S15. **i** Combined inhibition of SMG1 and DNA-PK reduces NRF2-S13 and -S40 phosphorylation in cells. Flag-tagged NRF2 was purified from 293 T cells treated with 200 μM DEM in the presence of NPD15008 and/or NU7441 for 2 h and analyzed by immunoblotting with phospho-specific antibodies. **j** Endogenous NRF2 was immunoprecipitated from HepG2 cells treated with 25 μM DEM for 3 h and probed with the indicated antibodies

Because phosphorylation of the SQ motifs at NRF2-S13 and -S40 is critical for NRF2 accumulation, we next examined whether other phosphatidylinositol 3-kinase–related kinases (PIKKs) function redundantly with SMG1. SMG1, ATM, ATR, and DNA-PK share substrate specificity for SQ motifs and are known to exhibit functional redundancy in cellular stress responses.^19,20,22,24–26^ We therefore investigated the contribution of these kinases to NRF2 activation under mild oxidative stress. In PC-3 cells exposed to mild oxidative stress, inhibition of DNA-PK using NU7441, a potent and selective DNA-PK inhibitor, markedly suppressed HO-1 protein expression, comparable to the effect of SMG1 inhibition by NPD15008, whereas inhibition of other SQ-directed PIKKs had minimal effects (Fig. 6a). NU7441 also dose-dependently reduced NRF2 and HO-1 protein levels under these conditions (Fig. 6b). In contrast to NPD15008, NU7441 did not affect UPF1 phosphorylation, indicating that DNA-PK inhibition does not impair NMD activity (Fig. 6b). Notably, combined inhibition of SMG1 and DNA-PK resulted in an additive suppression of NRF2 and HO-1 accumulation under mild oxidative stress (Fig. 6c), suggesting that SMG1 and DNA-PK function in a partially redundant manner. Consistent with these protein-level effects, NU7441 suppressed NRF2-dependent de novo transcription, with a modest additive effect observed upon co-treatment with NPD15008 (Fig. 6d, e). RT-qPCR analysis further confirmed reduced expression of NRF2 target genes, including HO-1 and GCLM (Supplementary Fig. 7a, b). Neither mild oxidative stress nor DNA-PK inhibition repressed NMD (Supplementary Fig. 7c, d). Both NPD15008 and NU7441 reduced de novo synthesis of NRF2 mRNA (Supplementary Fig. 7e), consistent with autoregulatory feedback control of NRF2 transcription. Induction of anti-ferroptotic gene expression, but not pro- or anti-apoptotic and necroptotic gene expression, in response to mild oxidative stress was suppressed by DNA-PK inhibition, with a modest additive effect upon co-treatment with NPD15008 (Supplementary Fig. 7f–i). Consistent with these findings, AZD7648, a structurally distinct DNA-PK inhibitor, also suppressed DEM-induced NRF2 accumulation (Supplementary Fig. 7j). DNA-PK inhibition also attenuated NRF2 accumulation induced by 2-AAPA and BSO (Supplementary Fig. 7k–n).

**Fig. 7:**
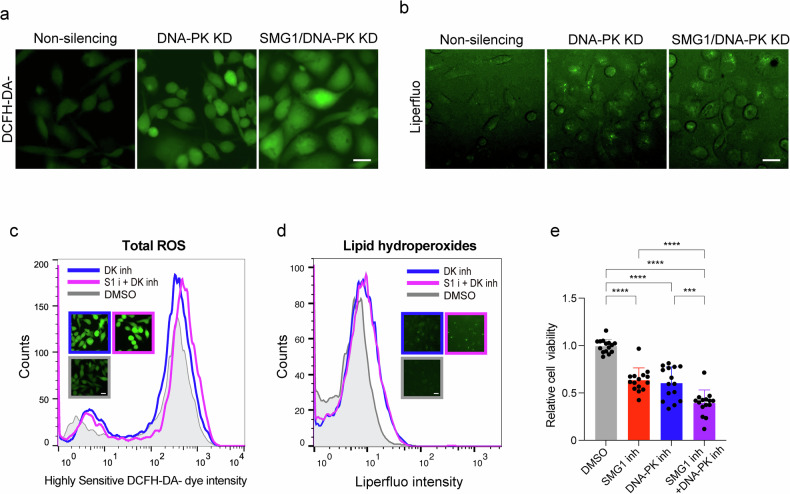
The SMG1-DNA-PK–NRF2 axis cooperatively suppresses ferroptosis. **a**, **b** PC-3 cells transfected with DNA-PKcs–targeting siRNA, with or without SMG1-targeting siRNA, for 48 h. Representative results from three biologically independent experiments are shown. Knockdown efficiencies of SMG1 and DNA-PKcs were confirmed by immunoblotting (see Supplementary Fig. 7o). **a** Intracellular total ROS levels measured using a photo-oxidation–resistant DCFH-DA probe. **b** Lipid hydroperoxide levels measured using the Liperfluo probe. **c**, **d** Flow cytometric analysis of PC-3 cells treated with control DMSO, 4 μM NU7441, or 4 μM NU7441 plus 6 μM NPD15008 for 24 h. Representative results from three biologically independent experiments are shown. Scale bar, 20 μm. **c** Intracellular total reactive oxygen species (ROS) levels measured using the DCFH-DA probe. **d** Lipid hydroperoxide levels measured using the Liperfluo probe. **e** Cell viability analysis of PC-3 cells treated with DMSO, 6 μM NPD15008, 4 μM NU7441, or their combination for 48 h. Cell viability was assessed using the alamarBlue assay. Data represent the mean ± SD from five technical replicates across two independent experiments. Statistical analysis was performed using one-way ANOVA with Tukey’s multiple-comparison test. *P < 0.05, **P < 0.01, ***P < 0.001, ****P < 0.0001

We next examined whether DNA-PK directly phosphorylates NRF2. In vitro kinase assays demonstrated that, similar to SMG1, DNA-PK directly phosphorylates NRF2 but not KEAP1 (Fig. 6f, g). DNA-PK exhibited a distinct substrate preference, preferentially phosphorylating NRF2 at S13 at levels comparable to those of the canonical substrate p53 at S15, whereas phosphorylation of NRF2 at S40 or S103 was less efficient and that at S410 was minimal (Fig. 6h). Combined inhibition of SMG1 and DNA-PK markedly reduced phosphorylation at both NRF2-S13 and NRF2-S40, whereas single inhibition had more modest effects (Fig. 6i). Endogenous NRF2 phosphorylation at S40 was readily detected in HepG2 cells treated with DEM and repressed by SMG1 and DNA-PK inhibition (Fig. 6j). Furthermore, pharmacological inhibition or genetic knockdown of DNA-PK, either alone or in combination with SMG1 inhibition or knockdown, increased intracellular ROS, lipid hydroperoxides, ferrous iron and 4-HNE levels and reduced cell viability (Fig. 7a–e and Supplementary Fig. 3c, 7o, p), without inducing apoptosis- or necroptosis-related marker proteins or the apoptotic marker annexin V (Supplementary Fig. 3c, 7q). To evaluate whether the dependency on the SMG1/DNA-PK–NRF2 axis extends beyond PC-3 cells, we examined HepG2 hepatocellular carcinoma cells. Pharmacological inhibition of SMG1, DNA-PK, or their combination increased intracellular ROS, lipid hydroperoxides, and ferrous iron accumulation in HepG2 cells (Supplementary Fig. 3r). SMG1 inhibition produced markedly stronger effects than DNA-PK inhibition alone, and the combination did not substantially exceed the effect of SMG1 inhibition alone, suggesting that SMG1 plays a dominant role in maintaining redox homeostasis in HepG2 cells. A modest increase in annexin V-positive cells was observed upon SMG1 inhibition in HepG2 cells, whereas this was not observed in PC-3 cells under equivalent conditions. Collectively, these results demonstrate that SMG1 and DNA-PK redundantly activate NRF2 signaling in response to mild oxidative stress. This coordinated activation is essential for maintaining redox homeostasis and promoting cell survival, a mechanism that may be particularly advantageous for cancer cells.

To assess the clinical relevance of the SMG1/DNA-PK–NRF2 axis in human cancers, we analyzed RNA-seq datasets from The Cancer Genome Atlas (TCGA). Among 87 NRF2 target genes identified in cultured cells,^50^ positive correlations with both SMG1 and PRKDC (DNA-PKcs) expression were observed in PRAD, PAAD, HNSC, BRCA, and UCEC (Supplementary Fig. 8a). In contrast, in LUAD, LUSC, and ESCA, NRF2 target gene expression showed a stronger correlation with DNA-PK than with SMG1 (Supplementary Fig. 8a). Given that SMG1- and DNA-PK-mediated phosphorylation of NRF2 weakens its interaction with the KEAP1 DLG motif, we examined whether oncogenic mutations in KEAP1 or NRF2, which constitutively activate NRF2 signaling, disrupt these correlations. Unexpectedly, oncogenic KEAP1 mutations in LUAD or NRF2 mutations in LUSC did not abolish the positive correlation between SMG1/DNA-PK expression and NRF2 target gene expression (Supplementary Fig. 8b). These findings suggest that NRF2 transcriptional output across multiple cancers is shaped by diverse upstream signaling inputs and cannot be explained solely by genetic alterations in the canonical KEAP1–NRF2 pathway.

## Discussion

Reactive oxygen species (ROS) play context-dependent roles in cancer biology, functioning not only as cytotoxic molecules but also as signaling mediators that promote tumor growth, metabolic adaptation, and therapy resistance. Oncogenic signaling, mitochondrial dysfunction, altered iron metabolism, and changes in redox enzyme activity collectively result in chronically elevated basal ROS levels in cancer cells. To survive in this pro-oxidant environment, cancer cells rely on finely tuned antioxidant systems to maintain redox homeostasis. Disruption of this balance can trigger ferroptosis and other forms of regulated cell death, positioning redox adaptation as a critical vulnerability in cancer. In this study, we identify SMG1 and DNA-PK as previously unrecognized upstream regulators of redox adaptation that determine cell fate in response to oxidative stress. Although the present study focuses on downstream signaling responses to oxidative stress, the precise subcellular origin and trafficking of ROS under these experimental conditions were not examined and will require dedicated future investigation.

Our data demonstrate that under mild oxidative stress, SMG1 and DNA-PK function cooperatively to promote cell survival by phosphorylating NRF2 at serine 40 and potentially at serine 13. These phosphorylation events weaken the interaction between NRF2 and its negative regulator KEAP1, leading to NRF2 accumulation and selective induction of antioxidant gene expression. Importantly, this NRF2 activation occurs without widespread engagement of anti-survival stress signaling pathways. Transcriptomic analyses of de novo–synthesized mRNAs revealed that pathways associated with growth arrest or cell death, including ATF4/CHOP, ATM–CHK2, and JNK/p38 signaling, remain largely inactive. This selective transcriptional response supports a model in which SMG1 and DNA-PK act as early sensors of redox imbalance, enabling cells to mount an adaptive antioxidant response before oxidative stress reaches cytotoxic levels. Our finding further indicates that SMG1/DNA-PK signaling precedes canonical ATF4-, ATM- and JNK/p38-dependent stress responses. Notably, PKC autophosphorylation was not detectably enhanced under these conditions, supporting a predominant role for SMG1/DNA-PK signaling in this context. The upstream molecular mechanism linking mild oxidative stress to SMG1 activation remains to be elucidated. Identifying the redox-sensing mechanism that connects early ROS accumulation to SMG1 activation will therefore be an important subject for future investigation.

Inhibition of either SMG1 or DNA-PK significantly attenuated NRF2 activation and antioxidant gene expression, leading to accumulation of total ROS, ferrous iron, and lipid hydroperoxides, ultimately sensitizing cells to ferroptotic death. These findings indicate that SMG1 and DNA-PK act in a partially redundant manner to safeguard NRF2-dependent antioxidant defenses under physiologically relevant oxidative conditions. This redundancy may reflect evolutionary pressure to ensure the robustness of redox protection. Consistent with this notion, comparative sequence analysis revealed conservation of SQ-directed PIKK phosphorylation motifs at either Ser13 or Ser40 of NRF2 across species. Most mammals retain both motifs, whereas reptiles and birds predominantly retain the S40-Q41 motif, and amphibians and fish preferentially retain the S13-Q14 motif (Supplementary Fig. 6a). These observations suggest that phosphorylation of either residue is sufficient to support NRF2 regulation by PIKKs, reinforcing the functional redundancy observed between SMG1 and DNA-PK.

KEAP1 cysteine residues are well-established sensors of electrophilic and oxidative stress and play a central role in canonical NRF2 activation.^11^ Although DEM has been reported to modify KEAP1 cysteines, several lines of evidence from our study argue that SMG1/DNA-PK-dependent NRF2 activation cannot be explained solely by canonical KEAP1 modification or degradation. First, neither DEM treatment nor SMG1 inhibition altered KEAP1 protein levels under low-dose oxidative stress conditions. Second, SMG1-dependent NRF2 accumulation was consistently observed across multiple oxidative stress paradigms, including BSO, 2-AAPA, rotenone, and arsenite, that do not directly target KEAP1 cysteines. Third, enforced expression of wild-type SMG1 was sufficient to increase NRF2 protein levels in the absence of exogenous oxidative stress, regulating NRF2 in parallel to KEAP1 sensing. Together, these findings suggest that SMG1 and DNA-PK regulate NRF2 either downstream of, or in parallel to, canonical KEAP1-mediated sensing. Our model is complementary to, rather than a replacement of, canonical KEAP1 cysteine sensing: SMG1/DNA-PK-mediated phosphorylation selectively weakens DLG-mediated KEAP1 binding while leaving the high-affinity ETGE-mediated anchoring intact. NRF2 activity is further shaped by KEAP1-independent degradation pathways, including the GSK3β–β-TrCP axis, which has been implicated in metabolic stress and growth factor withdrawal through association with the Neh6 domain.^11^ However, this pathway is generally associated with longer-term metabolic adaptation rather than acute oxidative stress sensing. In contrast, SMG1- and DNA-PK-mediated phosphorylation of NRF2 at Ser13 and Ser40, residues proximal to the KEAP1-binding DLG motif, represents a rapid and stress–intensity–dependent mechanism for NRF2 stabilization. This multi-layered regulatory architecture may explain why positive correlations between SMG1 or DNA-PK expression and NRF2 target gene expression are preserved even in tumors harboring oncogenic KEAP1 or NRF2 mutations.

A central conceptual advance of this study is the identification of a stress–intensity–dependent signaling switch that governs cell fate decisions in response to oxidative stress. Under mild oxidative stress, SMG1 and DNA-PK preferentially activate NRF2, thereby promoting redox homeostasis and cell survival. SMG1 kinase activity is preserved under these conditions and NMD remains active, thereby suppressing ATF4 mRNA accumulation and maintaining a pro-survival transcriptional state; this NMD-dependent restraint of ATF4 constitutes an additional survival mechanism operating in parallel with NRF2 activation. In contrast, when oxidative stress exceeds a critical threshold, this adaptive axis becomes insufficient and signaling is redirected toward anti-survival pathways, including ATF4, ATM–CHK2, and JNK/p38. Under excessive oxidative stress, we observed a modest reduction in KEAP1 levels concomitant with CHK2 activation, consistent with recent reports of ATM–CHK2–dependent KEAP1 modulation.^46^ This transition likely reflects coordinated signaling interplay among PIKKs. Specifically, DNA-PK deficiency has been reported to enhance ATM activation under excessive oxidative stress,^25^ and ATM–CHK2 signaling is known to engage p53-dependent transcriptional programs that antagonize NRF2.^51^ Concurrently, the integrated stress response is engaged, leading to eIF2α phosphorylation and translational reprogramming that allows selective translation of ATF4 through bypass of its inhibitory upstream open reading frames.^52^ Given that ATF4 and p53 can exert context-dependent antagonistic or cooperative effects on NRF2, simultaneous activation of these pathways may actively promote cell death once oxidative damage exceeds tolerable limits.^53,54^ Consistent with this model, a modest increase in annexin V-positive cells was observed upon SMG1 inhibition in HepG2 cells but not in PC-3 cells under equivalent conditions, suggesting that pharmacological collapse of the SMG1/NRF2 axis can push ROS accumulation beyond the ferroptotic threshold and into the apoptotic range in a cell-type-dependent manner.

From a therapeutic perspective, our findings highlight SMG1 as a particularly attractive target in cancer. An important implication of this study is that SMG1 inhibition may exert anticancer effects through multiple, mechanistically distinct pathways. Beyond its role in regulating NRF2-mediated redox homeostasis and ferroptosis resistance, SMG1 regulates nonsense-mediated mRNA decay (NMD) and Regnase-1-dependent inflammatory mRNA turnover.^28,55^ Accordingly, SMG1 inhibition may simultaneously destabilize intrinsic antioxidant defenses (NRF2 inhibition), enhance tumor immunogenicity by increasing aberrant transcript expression (NMD inhibition), and promote inflammatory signaling within the tumor microenvironment (Regnase-1 inhibition). Importantly, these three outputs are mechanistically independent, meaning that SMG1 inhibition can engage them simultaneously through a single molecular target. This convergence represents a unique combination of multiple anticancer mechanisms. Importantly, the ferroptosis-sensitizing effect of SMG1 inhibition described here is mechanistically distinct from NMD suppression, highlighting the versatility of SMG1 as a therapeutic vulnerability.

Notably, knockdown of core NMD components such as UPF1 would not constitute an appropriate experimental approach in this context, as it causes constitutive stabilization of NMD substrate mRNAs, including ATF4, thereby chronically pre-activating stress-response programs and confounding the interpretation of signaling responses.^30,32^ This limitation extends beyond the present study and broadly constrains the use of genetic NMD disruption in stress biology. Acute pharmacological inhibition circumvents this confound and was therefore employed here; DNA-PK inhibition was additionally used as an orthogonal, NMD-independent approach to confirm that NRF2 regulation reflects direct kinase activity rather than a secondary consequence of NMD impairment.

Although the present study is primarily based on cell-based analyses, independent SMG1 inhibitors have demonstrated antitumor efficacy in mouse models,^56^ and pharmacological NMD inhibition has recently advanced into early-phase clinical evaluation, further supporting the translational relevance of this axis. Because cancer cells rely on constitutively elevated SMG1/NRF2 signaling because of chronic oncogenic ROS production, a condition not equivalently sustained in normal tissues, pharmacological SMG1 inhibition may preferentially compromise tumor cell redox buffering while sparing normal cells that engage this axis only transiently. Dose optimization and intermittent dosing schedules, as well as combination with ROS-inducing therapies, represent rational strategies to exploit this therapeutic window in future translational studies.

In summary, this study identifies SMG1 and DNA-PK as key anti-ferroptotic kinases that directly phosphorylate and activate NRF2 to maintain redox homeostasis under mild oxidative stress. Together, these findings establish the SMG1/DNA-PK–NRF2 axis as an early redox-responsive signaling module that enables adaptive antioxidant responses under limited oxidative stress, while revealing a stress–intensity–dependent signaling hierarchy that governs the transition from redox adaptation to ferroptotic cell death. More broadly, the present findings establish SMG1 as a multifunctional stress-response kinase whose inhibition simultaneously disrupts NRF2-dependent redox buffering, NMD-mediated suppression of tumor neoantigen expression, and Regnase-1-dependent inflammatory mRNA decay to remodel the tumor microenvironment—three mechanistically independent outputs converging on a single molecular target.

## Materials and methods

### Plasmids, recombinant proteins, antibodies, siRNA and reagents

pEFh_SBP-SMG1 (residues 2–3661 of human SMG1, codon-optimized), pEFh_SMG8 (residues 1-991 of human SMG8, codon-optimized), pEFh_SMG9 (residues 1–520 of human SMG9, codon-optimized), pEFh_SBP-mTOR (residues 2-2549 of rat mTOR), pEFh_HA-mLST8 (residues 2–326 of human mLST8, codon-optimized), pEFh_SBP-ATM (residues 2-3056 of human ATM), pEFh_HA-ATMIN (residues 2–823 of human ATMIN), pEFh_SBP-ATR (residues 2–2644 of human ATR), pEFh_HA-ATRIP (residues 2–791 of human ATRIP), pEFs_SBP-Flag-NRF2 (residues 1–605 of human NRF2), pcDNA5_NRF2-HA_FRThyg (residues 1–605 of human NRF2), pGEX6p1_GST-NRF2 (residues 1–56 of human NRF2), pEFs_SBP-KEAP1 (residues 2–624 of human KEAP1), and pEFs_His-SBP-KEAP1-DC (residues 321–609 of human KEAP1) were constructed by cloning the corresponding cDNA fragments using standard molecular biology techniques. S13A, S40A, or S13A/S40A mutants of pcDNA5_NRF2-HA_FRThyg and S13E, S40E, or S13E/S40E mutants of pGEX6p1_GST-NRF2 (1–56) were generated using a standard site-directed mutagenesis protocol. GST-NRF2 14-mer peptide fusion proteins, designated as S13, S40, S103, and S410, were generated by inserting the corresponding oligonucleotides into the pGEX6p1 vector. pSR_Flag-SMG1, pSR_Flag-SMG1-DA, and pGEX6p1_GST-UPF1-S1096 plasmids were constructed as previously described.^57^ Detailed vector sequences are available upon request.

Recombinant proteins, including the SBP-SMG1:SMG9 complex, SBP-SMG1:SMG8:SMG9 complex, SBP-mTOR:HA-mLST8 complex, SBP-ATM:HA-ATMIN complex, SBP-ATR:HA-ATRIP complex, SBP-NRF2, SBP-KEAP1, and His-SBP-KEAP1-DC, were purified as previously described.^58^ Briefly, 3 × 10⁷ to 6 × 10⁸ HEK293T cells were co-transfected with the indicated plasmids using polyethylenimine (Polysciences). Two to three days after transfection, cells were lysed using a loose-fit Potter–Elvehjem homogenizer in NF buffer [20 mM Tris-HCl (pH 7.5), 150 mM NaCl, 0.25 M sucrose, 0.5% NP-40, 1% Tween 20, 1 mM dithiothreitol (DTT), protease inhibitor cocktail (Nacalai Tesque), EDTA-free phosphatase inhibitor cocktail (Nacalai Tesque)] supplemented with 50 μg/mL RNase A. SBP-tagged proteins were captured using streptavidin Mag Sepharose (GE Healthcare) by incubation at 4°C for 2 h with gentle rotation, followed by washing with NF buffer. Protein complexes were eluted at 4°C for 30 min using T buffer [20 mM HEPES-KOH (pH 7.5), 150 mM NaCl, 2.5 mM MgCl₂, 0.05% Tween 20] containing 2 mM DTT, EDTA-free protease inhibitor cocktail, phosphatase inhibitor cocktail, and 2 mM desthiobiotin (Sigma). For PIKKs, eluted proteins were dialyzed against PIKK kinase buffer [10 mM HEPES-NaOH (pH 7.5), 50 mM NaCl, 2.5 mM MgCl₂]. Purified DNA-PK was purchased from Promega (V5811).

GST fusion proteins, including GST-NRF2 (1–56), its mutants, GST-NRF2 (57–98), and GST-14-mer peptide fusion proteins, were purified using standard glutathione affinity chromatography with glutathione Sepharose 4B (GE Healthcare).

Antibodies against UPF1, phospho-S1078/S1096-UPF1 (clone 8E6), and phospho-T28-UPF1 were used as previously described.^59,60^ Clone 8E6 was used for AlphaScreen assays. Commercial antibodies used in this study included anti-phospho-T37/46-4EBP1 (236B4; CST, #2855), HO-1 (CST, #5853), eEF2 (CST, #2332), NRF2 (GeneTex, GTX103322; Proteintech, 80593-1-RR), phospho-S40-NRF2 (Abcam, #ab76026), KEAP1 (Millipore, MABS514), phospho-MLKL (Ser358; D6H3V; CST, #91689), caspase 3 (D3R6Y; CST, #14220), cleaved caspase 3 (5A1E; CST, #9664), GPx4 (Proteintech, 67763-01-Ig), CD71 (TfR1; Proteintech, 10084-2-AP), Flag (Sigma, F1804), and HA (Roche, 11867423001). An anti-phospho-S13-NRF2 antiserum was generated as described previously^35^ using a KLH-conjugated phospho-peptide [C-PPGLP(pS)QQDMD]. This antibody does not detect S13 phosphorylation for endogenous NRF2 due to antibody sensitivity.

The following siRNAs were used: SMG1 siRNA, GTGTATGTGCGCCAAAGTA; DNA-PK siRNA, Hs_PRKDC_8_HP Validated siRNA; and non-silencing control siRNA, AllStars Negative Control siRNA (QIAGEN). PIKK inhibitors were obtained from commercial sources: AZD8055 (mTORi; Sigma, ADV465749178), KU-55933 (ATMi; Sigma, SML1109), VE-821 (ATRi; Sigma, SML1415), NU7441 (DNA-PKi; Sigma, N1537), and AZD7648 (DNA-PKi; TargetMol, T7122). NPD15008 and compound 11j were synthesized by TLC Pharmaceutical Standards.

### Hybridoma production

Two 6-week-old female BALB/cAJcl mice were purchased from CLEA Japan. The Animal Care and Use Committee of Tohoku University (Permit number: 2022MdA-001) approved animal experiments. To develop mAbs against phospho-T3550-SMG1 [NTGQK(pT)QPDV], we intraperitoneally immunized two mice with 100 µg of the KLH-conjugated phospho-T3550-SMG1 peptides [C-NTGQK(pT)QPDV; Hokkaido System Science] plus Alhydrogel adjuvant 2% (InvivoGen). The procedure included three additional weekly immunizations (100 µg/mouse), which was followed by a final booster intraperitoneal injection (100 µg/mouse), two days before the harvest of spleen cells. Harvested spleen cells were subsequently fused with P3X63Ag8.U1 [P3U1; American Type Culture Collection (ATCC)] cells, using PEG1500 (Roche Diagnostics), after which hybridomas were grown in an RPMI-1640 medium (Nacalai Tesque) with 10% FBS (Thermo Fisher Scientific), 100 units/mL of penicillin, 100 μg/mL of streptomycin, and 0.25 μg/mL of amphotericin B (Nacalai Tesque). For the hybridoma selection, hypoxanthine, aminopterin, and thymidine (HAT; Thermo Fisher Scientific) were added into the medium. Supernatants were subsequently screened using enzyme-linked immunosorbent assay (ELISA) with the phospho-T3550-SMG1 and wild-type SMG1 (NTGQKTQPDV) peptides. To produce purified mAbs, hybridomas were cultured in Hybridoma-SFM (Thermo Fisher Scientific), and the purified mAbs were separated using Ab-Capcher (ProteNova).

### ELISA

The synthesized phospho-T3550-SMG1 and wild-type peptides were immobilized on Nunc Maxisorp 96 well immunoplates (Thermo Fisher Scientific) at 0.5 µg/mL concentration for 30 min at 37 °C. After washing with phosphate-buffered saline (PBS) containing 0.05% Tween 20 (PBST; Nacalai Tesque), wells were blocked with 1% bovine serum albumin (BSA)-containing PBST for 30 min at 37 °C. Plates were then incubated with supernatants of hybridomas, followed by peroxidase-conjugated anti-mouse immunoglobulins (1:2,000 diluted; Agilent Technologies). Next, enzymatic reactions were conducted, using ELISA POD Substrate TMB Kit (Nacalai Tesque), followed by measurement of the optical density at 655 nm using iMark microplate reader (Bio-Rad Laboratories).

### AlphaScreen assay for SMG1 kinase assay

AlphaScreen assay was performed on OptiPlate-384 (PerkinElmer) plates. 0.4 nM of SMG1 kinase (the SBP-SMG1:SMG9 complex) were incubated with 1 μM of the biotinylated UPF1-peptide [Biotin-PEG8-QIDVALSQDSTYQG (underline: S1096)] and 10 μM of ATP in SMG1 kinase buffer [10 mM HEPES-KOH (pH 7.5), 50 mM NaCl, 2.5 mM MnCl_2_ and 0.1% bovine serum albumin (BSA) and 0.1% Tween 20] at 26 °C for 120 min with or without 0.1 μL of 1 mg/mL of compounds (OCDD core: 9,600 compounds, NPDepo: 20,000 compounds, AIST synthetic compounds: 10560 compounds, AIST natural extract library isolated natural compounds: 2,240 compounds, and clinical development molecules: 4160 compounds) in a total volume of 10 μL per well. Then, the phosphorylated SMG1-substrated-peptides were labeled by the 312.5 μg/mL of anti-phospho-S1078/S1096-UPF1 (8E6) with 5 mM EDTA, which terminates kinase reaction, diluting in AlphaScreen detection buffer [10 mM Tris-HCl (pH. 7.0), 100 mM NaCl, 0.1% Tween 20 and 0.05% BSA] at 26 °C for 60 min in a total volume of 12 μL per well. After labeling, the mixture of streptavidin donor beads and protein A-acceptor beads (final concentration: 7.5 μg/mL) (PerkinElmer) were added in a total volume of 20 μL per well and incubated at 26 C for 16 to 19 h in the dark. After the incubation, laser excitations were carried out at 680 nm, and readings were performed at 520 to 620 nm using the EnVision Multilabel Reader (PerkinElmer). To determine the half maximal inhibitory concentration (IC_50_), 10^-9^ to 10^-4 ^M concentration of compounds were used.

### In vitro kinase assay

In vitro kinase assay of PIKK using radiolabeled ATP was performed as described elsewhere previously.^61^ In brief, recombinant PIKKs were incubated with the substrate peptides [UPF1: QIDVALSQDSTYQGRRRRR (underline: S1096), 4EBP1: KKKKKPQDYCTTPGGTLF (underline: T37), p53: KKKKKSVEPPLSQETFSD (underline: S15)] together with 16.6 to 49.8 nM of [γ-^32^P]ATP and 1 to 10 μM ATP in SMG1 kinase buffer with 0 to 50 μM of compounds at 30 °C for 15 min. The kinase reaction was terminated by heat inactivation at 65 °C for 15 min. After heat inactivation, each reaction mixture was spotted onto the center of a 1-cm square of P81 phosphocellulose paper (Millipore). Immediately immerse the paper into the 75 mM phosphoric acid in the glass beaker and gently shake for 5 min. Papers were transferred into new 75 mM phosphoric acid in the glass beaker and gently shaken for 5 min (repeat this washing procedure three times). After the final wash in phosphoric acid, transfer each paper to a new 1.5 mL microcentrifuge tube and measure radioactivity in the samples by Cerenkov counting without liquid scintillation fluid using the ‘^32^P program’ in a liquid scintillation counter.

### Luciferase-based NMD reporter assay

HeLa Tet-On Advanced cells were infected with pLenti6/FRT/eGFP-BSD lentivirus^62^ and single-cell clones were isolated by selection with 4 μg/mL of blasticidin S. Single integration of the Flp recombination target (FRT) site was confirmed by Southern blotting. pSV40-Luc2-21HBB_FRThyg_tk-Rluc (control reporter, NMD insensitive) or pSV40-Luc2-179HBB_FRThyg_tk-Rluc (test reporter, NMD sensitive) was integrated into the FRT site of HeLa Tet-On Advanced_FRT (clone #12) using Flp-In system (Thermo Fisher Scientific) to generate HeLa Tet-On Advanced-21H and HeLa Tet-On Advanced_-179H cells. Both reporters integrated into the same FRT site of HeLa Tet-On Advanced_FRT which ensures the same transcriptional rate of NMD-insensitive and -sensitive mRNA. For reporter assay, HeLa Tet-On Advanced cells were treated with 0.01 to 10 μM of NPD15008 for 16 h. Firefly luciferase activity which represents NMD inhibition and Renilla luciferase activity which represents cell number, were analyzed by Dual luciferase assay system (Promega). Details of reporter vector sequences are available upon request.

### Assay of cellular SMG1 inhibition

For phospho-T3550 of SMG1 analysis, the anti-phospho-T3550-SMG1 mouse monoclonal antibody (clone SpMab-7) was generated using a KLH-conjugated phospho-T3550 peptide as an antigen as described above. HEK293T cells were transfected with pEFh_SBP-SMG1 or an empty vector. For phospho-S1078/S1096 of UPF1 analysis, HeLa Tet-Off cells were treated with 0.01 to 30 μM of NPD15008 for 2 h. Cells were washed with PBS and then lysed in 1× SDS lysis buffer. Samples were analyzed by western blotting using anti-phospho-S1078/S1096-UPF1 (8E6) or anti-UPF1 antibody.

For analysis of the accumulation of natural NMD target mRNAs, HeLa Tet-Off cells were treated with 0.01 to 10 μM of NPD15008 for 4 h and harvested for total RNA isolation using RNeasy plus mini kit (QIAGEN). cDNA was synthesized using SuperScript VILO cDNA Synthesis Kit (Thermo Fisher Scientific). Reverse transcription- quantitative polymerase chain reaction (RT-qPCR) was performed using iCycler iQ Real-Time PCR Detection System (Bio-Rad Laboratories) with TaqMan gene expression assays probe (Thermo Fisher Scientific) and qPCR Master-mix (NIPPON GENE). mRNA expression was quantified and normalized to 18S ribosomal RNA. Mean values ± standard error represent data from more than three independent experiments. The following probes were used: SNHG1 (Hs00411543_m1); and 18S rRNA (Hs99999901_s1).

### Cell culture, transfection, viability assay, oxidative stress assay, and RT-qPCR assay

PC-3, a human prostate cancer cell line, was cultured in Ham’s F12 medium (Sigma-Aldrich) containing 10% (v/v) fetal bovine serum (FBS) and 1% (v/v) penicillin-streptomycin under a humidified atmosphere of 5% (v/v) CO_2_ at 37°C. HeLa Tet-Off and Hela Tet-On Advanced cell lines (Clontech), TIG-7, a normal human fibroblast cell line, HEK293T, a human embryonic kidney cell line expressing a mutant version of the SV40 large T antigen, were cultured in Dulbecco’s modified Eagle’s medium (DMEM) (Fujifilm Wako Pure Chemical) supplemented with 10% (v/v) FBS and 1% (v/v) penicillin-streptomycin under the same conditions.

Plasmid transfections were performed in six-well plates using polyethyleneimine (PEI; for HEK293T cells) or Lipofectamine 3000 (for PC-3 cells, Thermo Fisher Scientific) according to the manufacturer’s instructions. Cells were harvested or subjected to downstream analyses 42–48 h after transfection. siRNA transfections were performed in six-well plates using Lipofectamine RNAiMAX (Thermo Fisher Scientific) according to the manufacturer’s protocol. Cells were stimulated, harvested, or analyzed 42–48 h after siRNA transfection.

For the cell viability assay, cells were seeded at a density of 5 × 10^3^ cells per well in a 96-well half-well plate (Corning). After 24 h, the medium was replaced with fresh medium. The next day, the medium was replaced with a fresh medium containing either 0.2% (v/v) DMSO or 200 µM diethyl maleate (DEM) (Fujifilm Wako Pure Chemical, 041-21252) with or without 9 or 6 µM NPD15008 and/or 4 µM NU7441. After another 21 or 45 h, cells were incubated with alamarBlue reagent (Thermo Fisher Scientific) for 3 h. Fluorescence (excitation/emission: 555/590 nm) was measured by SpectraMax Paradigm plate reader with TUNE cartridge (Molecular Devices). As an alternative to alamarBlue assay, the number of viable cells was counted by analyzing the particle analysis of cell suspensions with ImageJ2 (Fiji) software.^63^

For oxidative stress treatment, cells were seeded at a density of 2.5 × 10^5^ cells per well in a 6-well plate and cultured for 2 days before assay. After 24 h of incubation, culture medium was changed. Cells were stimulated with DMSO or several oxidants, such as DEM, L-Buthionine-sulfoximine (BSO) (Sigma, B2515), 2-AAPA (Sigma, A4111), and rotenone (AdipoGen, AG-CN2-0516). At each time point, cells were washed in PBS and lysed with lysis buffer [2% LDS, 5 mM Tris-HCl (pH 6.8)] containing protease inhibitor cocktail and EDTA-free phosphatase inhibitor cocktail (Sigma-Aldrich), followed by homogenization using QIAshredder Mini Spin Column (QIAGEN).

For inhibition of PIKKs under oxidative stress, each PIKK inhibitor was added to a cell culture medium containing 200 µM DEM. The following compounds were used as PIKKs inhibitors: NPD15008 (SMG1i), KU-55933 (ATMi), VE-821 (ATRi), AZD8055 (mTORi) and NU7441 (DNA-PKi).

For analysis of mRNA expression under mild oxidative stress, PC-3 cells treated with oxidative stress described above were collected, and total RNA was purified using the RNeasy Plus Mini Kit (QIAGEN). cDNA was synthesized using the ReverTra Ace qPCR RT Master Mix (Toyobo). Real-time quantitative polymerase chain reaction (qPCR) was performed with a universal probe library (UPL) probe (Roche) and qPCR Master mix (NIPPON GENE) using iCycler iQ Real-Time PCR Detection System (Bio-Rad Laboratories) as described above. The following primer sets and probes were used: SNHG1 mRNA (TaqMan probe, Hs00411543_m1); HO-1 mRNA (primers: ggcagagggtgatagaagagg and agctcctgcaactcctcaaa, UPL probe #15); ATF4 mRNA (primers: tctccagcgacaaggctaa and ccaatctgtcccggagaa, UPL probe #76); SLC7A11 (primers: ccatgaacggtggtgtgtt and gaccctctcgagacgcaac, UPL probe #80); GCLM (primers: gacaaaacacagttggaacagc and cagtcaaatctggtggcatc, UPL probe #18) and 18S rRNA (TaqMan probe, Hs99999901_s1).

### Lentivirus production, concentration, and titer determination

We generated pLenti6-GFP_U6-sh-SMG1 and pLenti6-GFP_U6-sh-Rluc (siRNA target sequence: SMG1, AGGGATACAGTTGATATAT; Rluc, GGCCTTTCACTACTCCTAC) plasmids using standard molecular biology methods. The lentiviral supernatant was produced as follows. On the day of transfection, 5 × 10^6^ of 293FT cells were seeded into 10-cm culture dishes (20 dishes of each were prepared). Simultaneously, we prepared transfection mixture for each dish as the following method. Three µg of pLenti6-GFP_U6-sh-SMG1 or pLenti6-GFP_U6-sh-Rluc and 9 µg of ViraPower Lentiviral Packaging Mix (Thermo Fisher Scientific) were mixed in 1.5 mL of Opti-MEM medium (Thermo Fisher Scientific). Separately, 36 μL of Lipofectamine 2000 was diluted in another 1.5 mL of Opti-MEM. These two solutions were then mixed and incubated for 30 min at room temperature. After incubation, the mixtures were added to the 293FT cells, which were then cultured for 8 h. The medium was subsequently replaced for DMEM containing 10% FBS and the cells were cultured for 48 h. The culture supernatants were collected and filtered with a 0.22-µm Steriflip filter (Millipore) to generate the lentiviral supernatant. For the lentiviral concentration, we used PEG-it Virus Precipitation Solution (System Biosciences) following the manufacturer’s instructions.

To determine virus titers, we performed colony formation assay. In brief, 1 × 10^–3^ to 1 × 10^–5^ diluted lentivirus infection of 5 × 10^5^ of HT1080 cells cultured in 6-well plate was performed. Two days after infection, the medium was replaced with fresh medium containing 5 μg/mL blasticidin. After two weeks of culture, cells were stained with the crystal violet solution (1% crystal violet/10% ethanol) for 10 min at room temperature. After washing out the excess crystal violet with PBS, stained colonies were counted to determine the viral titer in transducing units (TU)/mL.

### In vivo xenograft analysis

For analysis effects of SMG1 on xenograft formation, PC-3 cell-derived xenografts were generated as follows. Nude mice (BALB/c, male) were inoculated bilaterally in the flanks with 7 × 10^6^ of PC-3 cells suspended in 0.1 mL of saline. One week after cell inoculation, when tumor nodules of approximately 5 mm in diameter had formed, lentivirus expressing shRNA targeted to SMG1 or non-silencing control (TU: 1 × 10^8^) was directly injected into the tumor nodule. Tumor volume was calculated as V = (L*S^2^)/2. The maximum (L) and minimum (S) diameters of the tumor were measured every week for 4 weeks after lentivirus infection. The tumor growth curves represent the mean relative tumor volume to day 0 ± SEM.

For analysis effects of NPD15008 on the xenograft formation, nude mice (BALB/c, male) were inoculated with 1 × 10^6^ of PC-3 cells on the right flank. Daily starting 3 days after inoculation, 100 μL of 12 mM NPD15008 in DMSO or 100 μL of DMSO control was administered daily by intraperitoneal injection. Body weight of each mouse was measured every other day from day 9 to day 23 of cell inoculation. Tumor volume was calculated same as lentivirus infected experiment. Due to the limited solubility of NPD15008 in DMSO (up to 12 mM), higher doses required larger volumes of DMSO, resulting in increased toxicity. Consequently, the amount of NPD15008 could not be increased. In compliance with the guidelines and standards for use of laboratory animals, mice exhibiting signs of distress, including excessive weight loss, were removed from the experiments.

For all xenograft analysis, mice were housed at the Animal Facility of Yokohama City University School of Medicine in accordance with the institutional guidelines and standards for use of laboratory animals. Prior to experiments, animal studies were reviewed and approved by the Animal Studies Committee of Yokohama City University (Permit number: F15-006).

### Western blot analysis, Immunoprecipitation assay and chromatin immunoprecipitation-qPCR analysis

For western blot analysis, samples were separated on a 4–20% gradient polyacrylamide gels (ATTO) in running buffer (250 mM Tris, 1920 mM glycine, 1% SDS) and transferred to Immobilon FL PVDF membranes (Merck) using EzFastBlot transfer buffer (ATTO). Signals were detected with the Luminata Classico Western HRP substrate (Merck), Luminata Forte Western HRP substrate (Merck) or ImmunoStar LD (Wako) and detected with a LuminoImager, LAS-4000, and Science Lab Image Gauge software (Fuji Photo Film). All experiments were performed at least three times, and representative results are shown.

For immunoprecipitation assay, 1 × 10⁶ cells were lysed by 0.5 mL in RIPA buffer [50 mM Tris-HCl, pH8.0, 150 mM Sodium Chloride, 0.5% Sodium Deoxycholate, 0.1% Sodium Dodecyl Sulfate, 1% NP-40, 50 μg/mL RNaseA, 1 mM dithiothreitol (DTT), protease inhibitor cocktail (Nacalai Tesque), EDTA-free phosphatase inhibitor cocktail (Nacalai Tesque)]. Lysates were incubated with antibodies [NRF2 (proteintech, 80593-1-RR Clone No.1I21), KEAP1 (proteintech, 10503-2-AP) or IgG control (CST, #2729)] and Dynabeads ProteinG (ThermoFisher) with gentle rotation at 4 °C for 2 h. The immunocomplexes were washed with washing buffer [50 mM Tris-HCl at pH 7.4, 50 mM NaCl, 0.05% Tween-20, 1 mM dithiothreitol (DTT), protease inhibitor cocktail (Nacalai Tesque), EDTA-free phosphatase inhibitor cocktail (Nacalai Tesque)], and boiled in 100 μL of standard 1 x LDS sample buffer and then analyzed by western blotting.

For chromatin immunoprecipitation assay, 1 × 10⁶ cells were cross-linked with 1% formaldehyde for 10 min at room temperature, followed by quenching with glycine, prior to being collected and lysed. Dynabeads ProteinA (ThermoFisher, 10002D) were pre-blocked with BSA and incubated overnight at 4 °C with 1 μg of either NRF2 antibody (proteintech, 80593-1-RR Clone No.1I21) or IgG control (CST #2729), and washed with RIPA buffer. Lysates containing chromatin fragments were incubated with washed antibody-bound beads for overnight at 4°C and washed with low-salt wash buffer [1% TritonX-100, 0.1% SDS, 2 mM EDTA (pH8.0), 150 mM NaCl, 20 mM Tris-HCl (pH8.0)], high salt wash buffer [1% TritonX-100, 0.1% SDS, 2 mM EDTA (pH8.0), 500 mM NaCl, 20 mM Tris-HCl (pH 8.0)] and LiCl buffer [0.25 M LiCl, 1% NP-40, 1% NaDOC, 1 mM EDTA, 10 mM Tris-HCl (pH 8.0)] with tube exchange at each steps. Immunoprecipitated protein-bound DNA fragments were eluted by elution buffer (1% SDS, 100 mM NaHCO3) and reverse cross-linked. DNA was purified using the FastGene Gel/PCR Extraction Kit (FastGene). Purified DNA was amplified and quantified by qPCR using TB Green® Premix Ex Taq™ II (Tli RNaseH Plus) (Takara). Total DNA served as input and quantified using the Qubit dsDNA HS Assay Kit (ThermoFisher). The following primer sets were used: HMOX1 (HO-1) enhancer (primers: GGTAGGCAGGAGGAAGTGAA and GGGCAGATTGAGGTGGACT); GCLM ARE (primers: GGAGAGCTGATTCCAAACTG and GAGTAACGGTTACGAAGCAC); p21 upstream (primers: GAGTCTTGCTCAGTGGGAGCTCTGGGAGTA and ATGTGACTTGGGGTGAGGCCTACTCGG).

### In vitro oxidative stress analysis

PC-3, a human prostate cancer cell line with a nonsense mutation in p53, was seeded at a density of 5 × 10^5^ cells per well in a 6-well plate. HepG2, a human hepatocellular carcinoma cell line with wild-type p53, was seeded at a density of 2 × 10^6^ cells per 10 cm dish. After 24 h, culture medium was replaced with freshly prepared medium containing DMSO, diethyl maleate (DEM) (Fujifilm Wako Pure Chemical), NPD15008, or NU7441 as indicated. After an additional 24 h, cells were subjected to analysis. Intracellular total ROS, lipid peroxides, ferrous iron and annexin V apoptosis marker were detected with DCFH-DA probe (Dojindo), Liperfluo (Dojindo), FerroOrange (Dojindo), and Annexin V-633 Apoptosis Detection Kit (Nacalai Tesque), respectively. Fluorescence was detected on an EVOS FL fluorescence microscope (AMG), FACSCalibur (BD Biosciences), or LSR Fortessa (BD Biosciences). For flow cytometry analysis, cells were suspended in phenol red-free DMEM following detachment with Accutase (Innovative Cell Technologies). Flow cytometry data were analyzed by FlowJo (BD Biosciences).

### In vivo oxidative stress analysis

For in vivo analysis of the effects of NPD15008 on apoptosis and ferroptosis, C57BL/6 mice (female, 6–8 weeks) were intraperitoneally injected with either 100 uL of 9 mM NPD15008 or 100 μL of DMSO as a vehicle control. At 6 or 8 h after injection, the livers were collected and mechanically dissociated by gentle tearing in RPMI 1640 containing 0.5 mM collagenase type IV (Sigma-Aldrich), followed by filtration through a 70-μm cell strainer. The cell suspension was centrifuged at 50 x *g* for 5 min, and the pellet was washed once with Hanks’ Balanced Salt Solution (HBSS). Cells were stained with Annexin V-633 Apoptosis Detection Kit (Nacalai Tesque), DCFH-DA (Dojindo), Liperfluo (Dojindo), and FerroOrange (Dojindo). Stained cells were analyzed by flow cytometry and fluorescence intensity was quantified as the mean fluorescence intensity (MFI). Flow cytometric analysis was performed using an LSR Fortessa (BD Biosciences) and all data were analyzed using FlowJo software (Tree Star). For all apoptosis and ferroptosis analyses, mice were housed at the Animal Facility of Kindai University in compliance with the institutional guidelines and standards for use of laboratory animals (KAPS-2025-002).

### Definition of mild and excessive oxidative stress

To experimentally distinguish between mild and excessive oxidative stress, intracellular total ROS levels were estimated using DCFH-DA-based fluorescence analysis as a semi-quantitative readout of total ROS accumulation. DCFH-DA detects multiple reactive oxygen species, including hydrogen peroxide, peroxyl radicals, and related oxidants, and was therefore used to assess intracellular total ROS rather than specific ROS. Oxidative stress was induced by diethyl maleate (DEM), which depletes intracellular glutathione and thereby promotes endogenous ROS accumulation. For calibration purposes, cells were additionally exposed to defined concentrations of exogenously supplied hydrogen peroxide (0–100 μM) prior to DCFH-DA staining. Because basal intracellular ROS levels under steady-state conditions are estimated to be below approximately 100 nM,^64^ their contribution to the fluorescence signal was considered negligible relative to exogenously applied H₂O₂ and DEM-induced ROS accumulation. Under these conditions, treatment with 200 μM DEM for 4 h resulted in intracellular total ROS levels comparable to those observed upon exposure to at or below 1 μM H₂O₂, without activation of canonical anti-survival stress pathways such as ATM–CHK2 or JNK signaling. We operationally defined this condition as mild oxidative stress. In contrast, DEM concentrations ≥800 μM were required to induce intracellular total ROS levels comparable to those observed following approximately 100 μM H₂O₂ and were accompanied by robust phosphorylation of CHK2 and JNK. We operationally defined this condition as excessive oxidative stress. These definitions were based on functional signaling outcomes rather than absolute ROS quantification.

### Microscale thermophoresis analysis

For a quantitative measurement of the equilibrium dissociation constant, *K*_D_ value, between KEAP1-DC and NRF2 (1–56), microscale thermophoresis (MST) experiments were performed. His-SBP-KEAP1-DC (residues 321–609), used as the MST target, was fluorescently labeled using the Monolith NT His-Tag Labeling Kit (NanoTemper Technologies). GST-NRF2 (1–56) wild-type (WT) or mutants (S13E, S40E and S13E/S40E) used as MST ligands, were serially diluted in twofold steps from a 63 μM stock solution in PBS containing 1 mM DTT.

A solution of 100 nM labeled His-SBP-KEAP1-DC was mixed with each diluted GST-NRF2 (1–56) WT or mutants in a 1:1 ratio. The final concentration of labeled His-SBP-KEAP1-DC was 50 nM, and the final concentrations of GST-NRF2 (1–56) ranged from 31.5 μM to 0.953 μM. Mixtures were incubated at 4 °C for 1 h to reach an equilibrium state. Each sample was loaded into glass capillaries (NanoTemper) and the thermophoresis analysis was performed using a Nanotemper Monolith NT.115 instrument (40% Excitation power and Medium MST power). The MST curves and *K*_D_ values were derived from two independent experiments using the NanoTemper analysis software (NanoTemper).

### 4sU metabolic RNA labeling

PC-3 cells were seeded in a 6-well plate at 2.5 × 10^5^ cells per well. After 24 h, the medium was replaced with 2 mL of fresh medium. The next day, the medium was quickly replaced with a freshly prepared medium containing 200 µM 4-thiouridine (4sU) with either 0.2% (v/v) DMSO or 200 µM DEM with or without 9 µM NPD15008 and/or 4 µM NU7441. The cells were incubated at 37 °C for 3 h and lysed in 500 µL of ISOGEN II (NIPPON GENE) supplemented with 1 mM DTT, and the prepared sample solution was stored at –20 °C. The manufacturer’s instructions were followed for RNA extraction using ISOGEN II, and the RNA samples were assessed for quality and quantity on MultiNA capillary electrophoresis instrument (Shimadzu).

### SLAMseq and QuantSeq

Total RNAs were processed according to the standard SLAMseq protocol described previously.^65^ 5 µg of total RNA was incubated in 50 µL of alkylation buffer [50 mM NaPO_4_ buffer (pH 8.0), 50% (v/v) DMSO, 10 mM iodoacetamide] at 50 °C for 15 min. The reaction was quenched by adding 1 µL of 1 M DTT, followed by ethanol precipitation. 500 ng of total RNA was used as an input for QuantSeq 3’ mRNA-Seq Library Prep Kit FWD for Illumina (Lexogen). The cDNA library was prepared according to the manufacturer’s instructions. Libraries were assessed for quality using a MultiNA capillary electrophoresis instrument (Shimadzu), multiplexed to equimolar concentrations, and sequenced using the HiSeq X system (Illumina) in PE-150 mode.

### Bioinformatic analysis

4sU-labeled nascent RNAs were quantified using SLAM-DUNK v 0.4.2 with default parameters.^65,66^ 12 bases from the 5’ end were trimmed as adaptor-clipped reads, and then five or more subsequent adenines from the 3’ end were regarded as the remaining poly (A) tail and removed. Up to 100 regions with multiple mapped reads were allowed. The sequence reads were aligned on genome-wide 3’ UTR sequences generated based on the human genome sequence (GRCh38.p13) and annotation data. Differential gene expression analysis was performed using DESeq2 v 1.3.8.^67^ To analyze the nascent T > C reads with the DESeq2, we calculated size factors using the total read counts. The log_2_ fold changes calculated by DESeq2 were normalized as z-scores and used to plot the cumulative curves. The following gene sets were used to plot cumulative curves: NRF2 transcriptional target genes^50^; ATF4/CHOP transcriptional target genes^68^; genes involved in pro-ferroptosis and anti-ferroptosis^46^ ; FOXO1 (CHEA Transcription Factor Targets), FOXO3a (GeneRIF Biological Term Annotations), and NF-κB (GeneRIF Biological Term Annotations) transcriptional target genes, as well as genes involved in anti-apoptosis and necroptosis from Harmonizome^69^; p53 (M5939) and AP-1 (M7477) transcriptional target genes, as well as genes involved in pro-apoptosis from GSEA MSigDB 3.0.^70,71^

TCGA RNA-seq datasets [prostate adenocarcinoma (PRAD), pancreatic adenocarcinoma (PAAD), head and neck squamous cell carcinoma (HNSC), breast invasive carcinoma (BRCA), uterine corpus endometrial carcinoma (UCEC), lung adenocarcinoma (LUAD), lung squamous cell carcinoma (LUSC), and esophageal carcinoma (ESCA)] were downloaded from NCI Genomic Data Commons Data Portal (https://portal.gdc.cancer.gov/v1/). Count data were normalized using size factors obtained by DESeq2 and used to calculate Spearman *R*.

For the mutation analysis, LUAD and LUSC samples were categorized into two groups based on the status of KEAP1 and NFE2L2 mutations, respectively. Mutation data and functional annotations were obtained from the TCGA Pan-Cancer Atlas project.^72^ Samples harboring mutations annotated as “Oncogenic”, “Likely Oncogenic”, or “Resistance” were assigned to the “harmful” group, while all other samples were classified as the “no harm” group. Spearman correlation analysis was performed independently for each group to evaluate whether the presence of these mutations influenced the association between SMG1 or DNA-PK expression and NRF2-target genes expression.

### Statistical analysis

Statistical analyses were performed using one-way ANOVA with Tukey’s multiple-comparison test, Sidak’s multiple-comparison test or unpaired *t* test. Data were obtained through three or four independent experiments and values represent the means ± standard error or standard deviation. *P* values < 0.05 were considered statistically significant.

## Supplementary information

Supplementary Materials

Supplementary Table 2

Supplementary Table 3

Supplementary Table 4

Supplementary Table 1

## Data Availability

All deep sequencing data from this study are publicly available in DDBJ (DRA017003). Additional information required to reanalyze the data reported in this work paper is available from the lead contact upon request.

## Code availability

All custom code used in this study is available from the lead contact upon request.
