# Supplementary material for: Two stress-responsive kinases suppress ferroptosis by activating antioxidant programs under mild oxidative stress
Source: Signal Transduct Target Ther. 2026 Aug 3;11:306. doi: 10.1038/s41392-026-02892-1 (PMC13429692; doi:10.1038/s41392-026-02892-1)
Supplement: Supplementary file 1 — Supplementary Materials [file 41392_2026_2892_MOESM1_ESM.docx]

**Supplementary data for**

**Two stress-responsive kinases suppress ferroptosis by activating antioxidant programs under mild oxidative stress**

Yumiko Fujikawa, Hirotatsu Imai, Tetsuo Onuki, Kouji Hoshino, Marco A. De Velasco, Kazuhiko Matsuo, Hitomi Kurosawa, Kyoko Aoyagi, Hiroko Hirose, Yoshie Nakamura, Akiko Uchiyama, Kae Suzuki, Mariko Mizuguchi, Hidehisa Takahashi, Hiroyuki Osada, Noritaka Kagaya, Kazuo Shin-ya, Hiroyuki Satofuka, Yukinari Kato, Hidehito Kuroyanagi, Daisuke Utsumi, Kenzo Takahashi, Takashi Nakayama, Hirotsugu Uemura, Hiroji Uemura, Minoru Yoshida, Shigeo Ohno*, Akio Yamashita*

*Corresponding author: Akio Yamashita, Shigeo Ohno,

**Email:** yamasita@phar.kindai.ac.jp, ohnos@juntendo.ac.jp

**This word file includes:**

Figure legends for Supplementary Fig. 1 to 8 and Supplementary Table 1 to 4

References

**Other materials for this manuscript include:**

Supplementary Fig. 1 to 8, which are uploaded separately as TIFF files

Supplementary Tables 1 to 4, which are uploaded separately

**Figure legends**

**
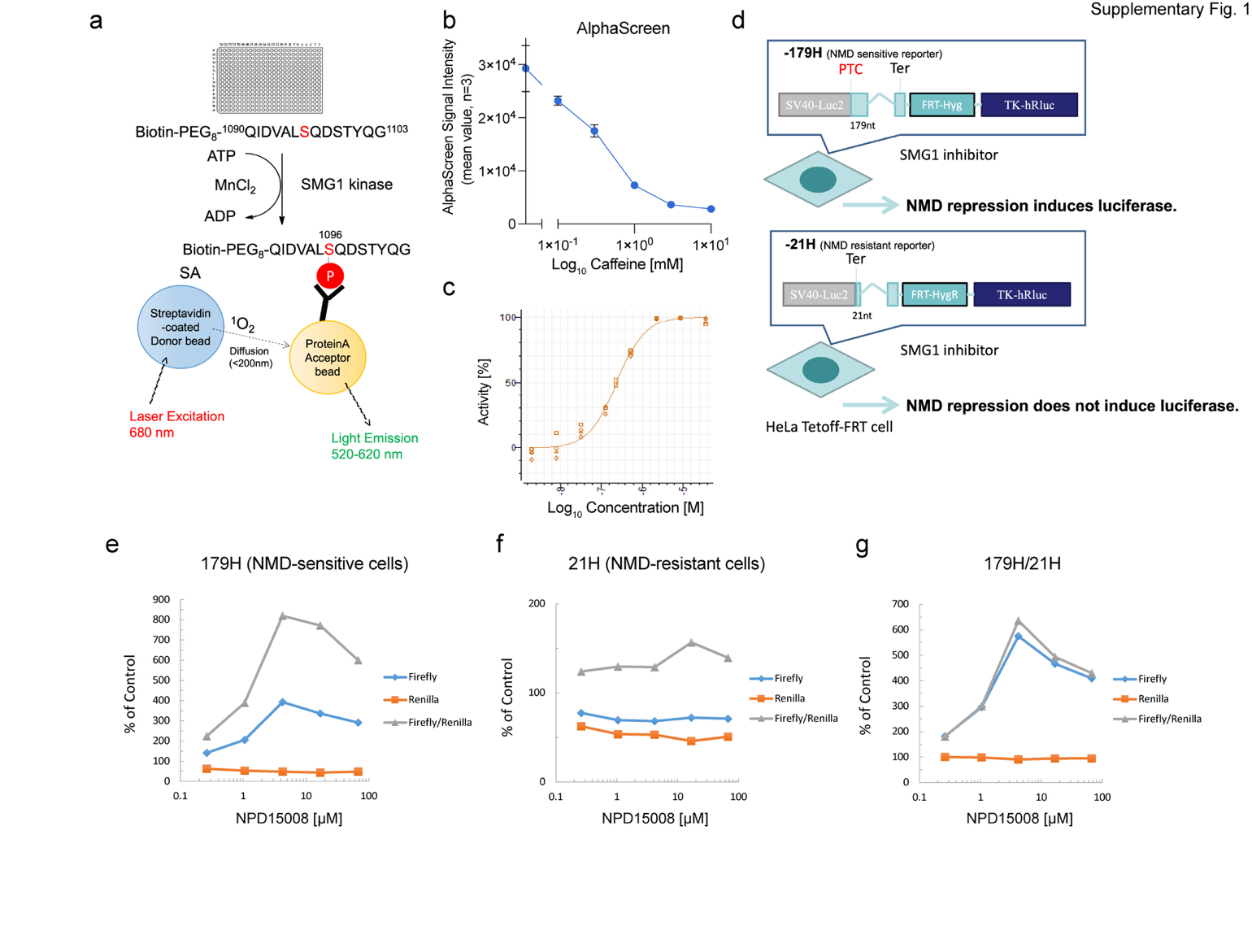
**

**Supplementary Fig.1**

Identification of SMG1 inhibitor, NPD15008. **a** Schematic illustration of the AlphaScreen-based high-throughput screening assay for SMG1 inhibitors. Compounds were spotted onto 384-well assay plates, and 0.1 μL of each compound (1 mg/mL stock in DMSO) was added per well. **b** AlphaScreen-based analysis of SMG1 kinase activity. Purified SMG1, UPF1 substrate peptide, and ATP were incubated with the indicated concentrations of caffeine at room temperature for 2 h. AlphaScreen luminescence signals were detected using an anti–phospho-Ser1096-UPF1 antibody. Data are representative of three independent experiments and are shown as the mean ± standard error; the graph displays mean values from two independent experiments. **c** Determination of the IC₅₀ value for inhibition of SMG1 kinase activity by NPD15008 using the AlphaScreen assay. Data are from three independent experiments and are shown as the mean ± standard error. **d** Luciferase-based nonsense-mediated mRNA decay (NMD) reporter assay to evaluate SMG1 inhibition. A schematic illustration of the NMD reporter construct is shown. Stable reporter cell lines were generated using the Flp-In system. Cells harboring an NMD-sensitive reporter (179H) (**e**) or an NMD-resistant reporter (21H) (**f**) were treated with the indicated concentrations of NPD15008 for 24 h. Firefly luciferase activity was normalized to Renilla luciferase activity and to untreated controls. **g** For the NMD-sensitive reporter, luciferase activity was additionally normalized to the NMD-resistant reporter signal.

**
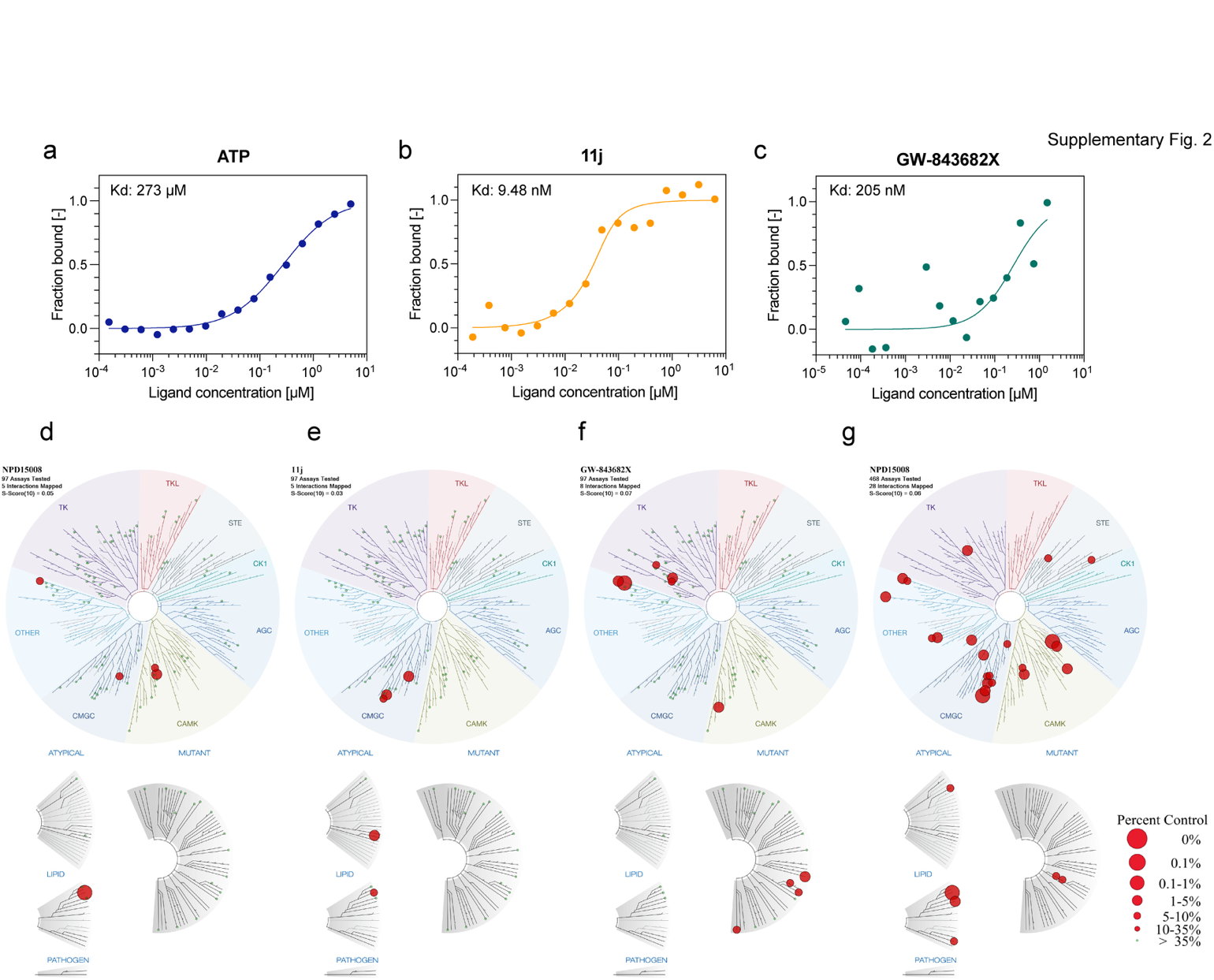
Supplementary Fig. 2**

Affinity and specificity of SMG1 inhibitory compounds. (**a**–**c**) Binding affinities of ATP (**a**), Pfizer compound 11j (**b**), and GW-843682X (**c**) for the SMG1–SMG9 complex. Microscale thermophoresis (MST) analyses were performed using fluorescently labeled SMG1 and increasing concentrations of the indicated compounds. Representative binding curves from single experiments are shown, and corresponding *K_D_* values are indicated. **d**–**g** Kinase selectivity profiles against 97 conventional kinases by NPD15008 (**d**), Pfizer compound 11j (**e**), and GW-843682X (**f**), and against 468 kinases by NPD15008 (**g**) were analyzed using the KINOMEscan platform (DiscoverX). Data were visualized using TREEspot software. Red circles indicate kinases affected by the indicated compounds. Values represent relative recovery of each kinase on ATP-affinity beads in the presence of 10 μM compound (e.g., 100 indicates no effect; 10 indicates 90% inhibition), with a 35% inhibition threshold indicated. Raw data are provided in Supplementary Table 2.

**
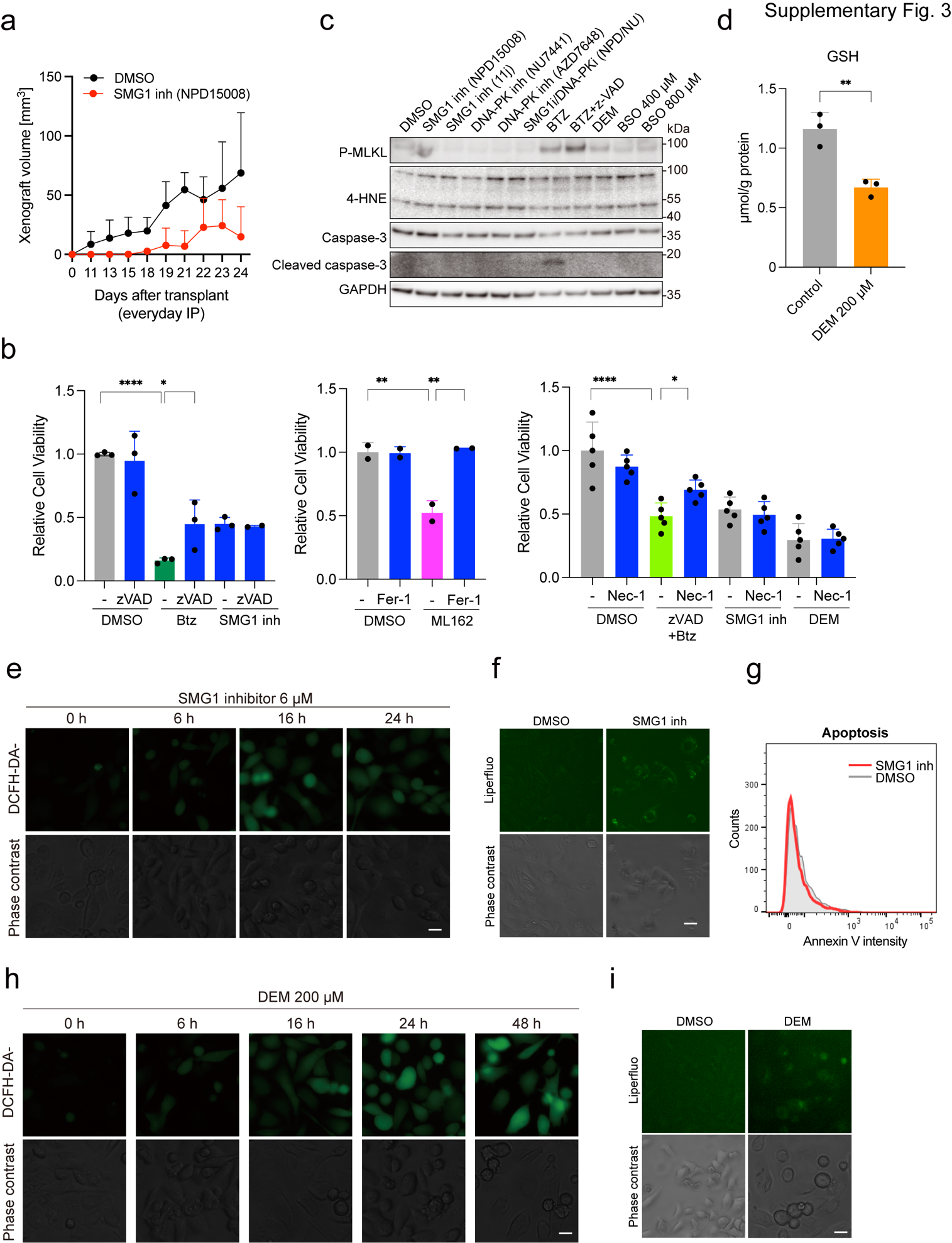
Supplementary Fig. 3**

NPD15008 promotes intracellular ROS accumulation and lipid hydroperoxidation. **a** PC-3 xenograft growth in nude (nu/nu) mice treated with NPD15008. Mice were subcutaneously inoculated with PC-3 cells and treated by intraperitoneal injection with either NPD15008 (12 mM in 100% DMSO, 100 μL per mouse; n = 5) or vehicle control (100 μL of 100% DMSO; n = 5), starting 3 days after tumor inoculation. Tumor volumes were measured from day 9. Data are shown as mean ± standard error. **b** Effects of cell death pathway inhibitors on NPD15008-induced loss of cell viability. PC-3 cells were treated with 6 μM NPD15008 (SMG1 inhibitor), 0.1 μM bortezomib (Btz; apoptosis inducer), or 0.3 μM ML162 (GPX4 inhibitor; ferroptosis inducer), in the presence or absence of 40 μM Z-VAD-FMK (zVAD; pan-caspase inhibitor), 50 μM necrostatin-1 (Nec-1; necroptosis inhibitor), or 5 μM ferrostatin-1 (Fer-1; ferroptosis inhibitor), as indicated, for 24 h. Combined treatment with bortezomib and Z-VAD-FMK induces necroptosis that is sensitive to necrostatin-1 ^1^ . Cell viability was assessed using the alamarBlue assay. Data are shown as mean ± standard error from five technical replicates derived from three (left) or two (middle and right) biologically independent experiments. Statistical analysis was performed using one-way ANOVA with Tukey’s multiple-comparison test. *P<0.05, **P<0.01, ***P<0.001, ****P<0.0001 **c** Immunoblot analysis of apoptosis- and necroptosis-related markers in PC-3 cells treated for 24 hours with SMG1 inhibitors (NPD15008 or 11j), DNA-PK inhibitors (NU7441 or AZD7648), bortezomib (Btz), Btz plus Z-VAD-FMK, DEM, or BSO (Glutamate-cysteine ligase inhibitor). Total cell extracts were probed with the indicated antibodies. **d** Intracellular glutathione (GSH) levels in PC-3 cells treated with 200 μM DEM for 4 h. Data are from three independent experiments and are shown as mean ± standard error. Statistical significance was determined using Student’s t-test. **P < 0.01. **e** Intracellular total ROS levels detected using the DCFH-DA (2′,7′-dichlorodihydrofluorescein diacetate) probe in PC-3 cells treated with 6 μM NPD15008 for the indicated time points. Scale bar, 20 μm. **f** Lipid peroxide levels detected using the Liperfluo probe in PC-3 cells treated with 6 μM NPD15008 for 24 h. Scale bar, 20 μm. **g** Flow cytometric analysis of annexin V staining to assess apoptosis in PC-3 cells treated with or without NPD15008 for 24 h. **h** Intracellular total ROS levels detected using the DCFH-DA probe in PC-3 cells treated with 200 μM DEM for the indicated time points. Scale bar, 20 μm. **i** Lipid peroxide levels detected using the Liperfluo probe in PC-3 cells treated with 200 μM DEM for 24 h. Scale bar, 20 μm. Representative results from three biologically independent experiments are shown unless otherwise indicated.

**
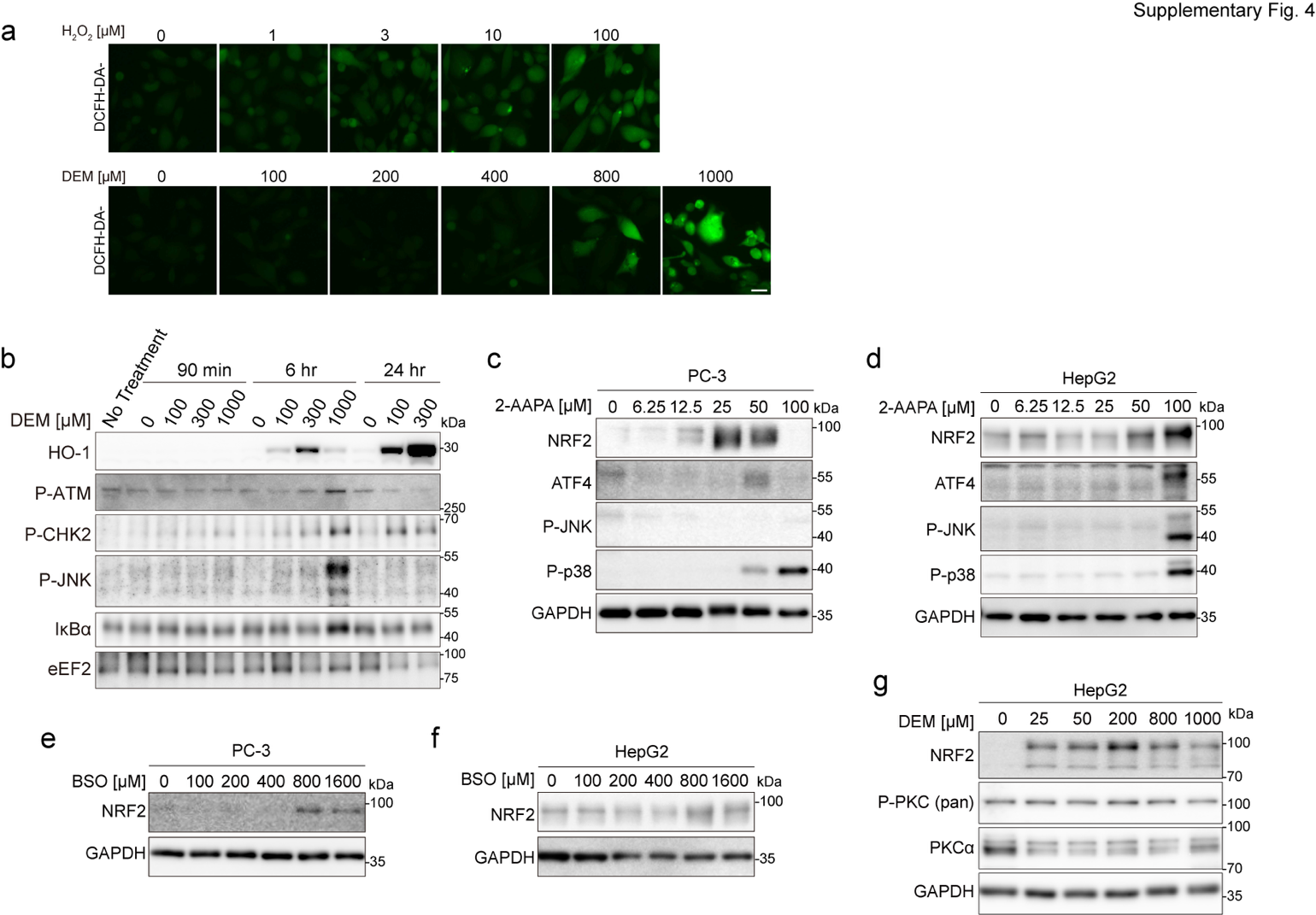
**

**Supplementary Fig. 4**

Dose- and time-dependent effects of oxidants on redox signaling. **a** Intracellular total ROS levels detected using the DCFH-DA probe in PC-3 cells treated with the indicated concentration of H_2_O_2_ for 30 min (top) or DEM for 4 h (bottom). Scale bar, 20 μm. **b** Time- and dose-dependent effects of DEM on pro-survival and anti-survival signaling proteins in PC-3 cells. Cells were treated with 0, 100, 300, or 1000 μM DEM for 1.5, 6, or 24 h. Samples were not available for 1000 μM DEM at 24 h due to complete cell detachment. Total cell extracts were analyzed by immunoblotting. eEF2 served as a loading control. Blots are representative of three biologically independent experiments. **c**, **d** Effects of 2-AAPA on redox signaling in PC-3 cells (**c**) and HepG2 cells (**d**). Cells were treated with 0, 6.25, 12.5, 25, 50, or 100 μM 2-AAPA for 4 h (PC-3) or 3 h (HepG2). Total cell extracts were analyzed by immunoblotting with the indicated antibodies. GAPDH served as a loading control. **e**, **f** Effects of BSO on redox signaling in PC-3 cells (**e**) and HepG2 cells (**f**). Cells were treated with 0, 100, 200, 400, 800, or 1600 μM BSO for 3 h (PC-3) or 4 h (HepG2). Total cell extracts were analyzed by immunoblotting with the indicated antibodies. GAPDH served as a loading control. Representative results from three biologically independent experiments are shown unless otherwise indicated. **g** Effects of DEM on PKC autophosphorylation in HepG2 cells. Cells were treated with 0, 25, 100, or 1000 μM DEM for 3 h. Total cell extracts were analyzed by immunoblotting with the indicated antibodies. GAPDH served as a loading control.

**
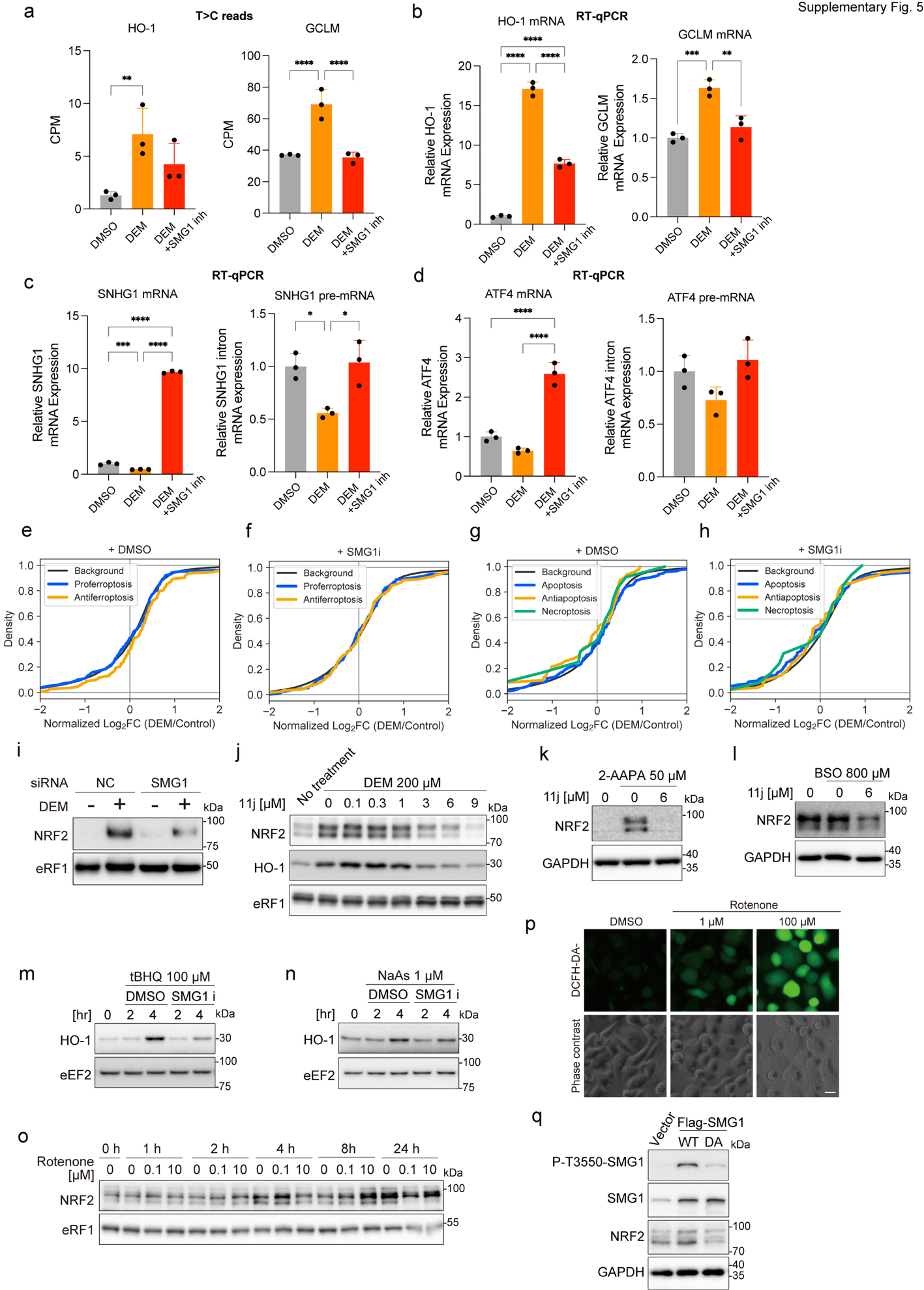
Supplementary Fig. 5**

SMG1 kinase activity selectively promotes NRF2 signaling under mild oxidative stress. **a** Counts per million (CPM) values of selected NRF2 target genes (HO-1 and GCLM) derived from SLAM-seq analysis of PC-3 cells treated with 200 μM DEM with or without NPD15008 for 3 h. **b** Relative mRNA levels of NRF2 target genes (HO-1 and GCLM) measured by RT–qPCR and normalized to 18S rRNA in PC-3 cells treated with 200 μM DEM in the presence or absence of NPD15008 for 4 hours. **c**, **d** Relative mRNA levels of spliced mRNAs and introns of selected NMD targets, SNHG1 (**c**) and ATF4 (**d**), normalized by 18S rRNA in response to 200 μM DEM with or without NPD15008 (SMG1 inhibitor) for 4 hours in PC-3 cells. mRNAs were analyzed by RT-qPCR. **e**, **f** Cumulative distribution of log₂ fold changes in de novo-transcribed mRNAs for selected sets involved in pro-ferroptosis (blue) and anti-ferroptosis (orange) pathways relative to all expressed genes (black). PC-3 cells were treated with 200 μM DEM alone (**e**) or in combination with 9 μM NPD15008 (**f**) for 3 hours. Statistical significance was assessed using adjusted Welch’s t-tests. Adjusted Welch's t-test P-values: pro-ferroptosis genes (*P* = 1.0 in (**e**) and *P* = 0.74 in (**f**)), and anti-ferroptosis genes (*P* = 0.01 in E and *P* = 0.55 in (**f**)). **g**, **h** Cumulative distribution of log₂ fold changes in de novo transcribed mRNAs for gene sets involved in pro-apoptosis (blue), anti-apoptosis (orange), and necroptosis (green) pathways, relative to all expressed genes (black). PC-3 cells were treated with 200 μM DEM alone (**g**) or in combination with 9 μM NPD15008 (**h**) for 3 hours. Statistical significance was assessed using adjusted Welch’s t-tests. Adjusted Welch's t-test P-values: pro-apoptosis genes (*P* = 1.0 in (**g**) and *P* = 1.0 in (**h**)), anti-apoptosis genes (*P* = 0.18 in (**g**) and *P* = 1.0 in (**h**)), and necroptosis genes (*P* = 1.0 in (**g**) and *P* = 0.95 in (**h**)). **i** SMG1 knockdown suppresses DEM-induced NRF2 protein accumulation in PC-3 cells. PC-3 cells transfected with SMG1-targeting or control siRNA. 48-hour post transfection, cells were treated with or without 200 μM DEM. Total cell extracts were probed with the antibodies indicated. eRF1 was probed as a loading control. **j**–**l** Effect of SMG1 inhibitor 11j on DEM-, 2-AAPA-, or BSO-induced NRF2 accumulation. PC-3 cells were treated with 200 μM DEM (**j**), 25 μM 2-AAPA (**k**), or 800 μM BSO (**l**) in the presence or absence of 11j for 4 hours. Whole-cell lysates were analyzed by immunoblotting. eRF1 or GAPDH served as loading controls. **m**, **n** Effect of SMG1 inhibitor NPD15008 on tert-butylhydroquinone (tBHQ)- or sodium arsenite (NaAs)- induced NRF2 target HO-1 accumulation. PC-3 cells were treated with 100 µM tBHQ (**m**) or 1 µM NaAs (**n**) in the presence or absence of NPD15008. Whole-cell lysates were analyzed by immunoblotting. eEF2 served as a loading control. **o** NRF2 accumulation following treatment with 0.1 or 10 μM rotenone for the indicated times. Total cell extracts were probed with the antibodies indicated. eRF1 was probed as a loading control. **p** Intracellular total ROS in HepG2 cells treated with rotenone at the indicated concentrations for 3 h, detected using the DCFH-DA probe. Scale bar, 20 μm. **q** Effect of SMG1 wild type or kinase-inactive mutant (DA: D2335A) over-expression on NRF2 expression. HEK293T cells were transfected with Flag-tagged wild-type SMG1, the kinase-dead D2335A mutant, or an empty vector. Whole-cell lysates were collected 48 h after transfection and analyzed by immunoblotting with the indicated antibodies. Same samples of Fig. 3k were used. Unless otherwise indicated, data represent mean ± SEM from more than three independent experiments. *P<0.05, **P<0.01, ***P<0.001, ****P<0.0001

**
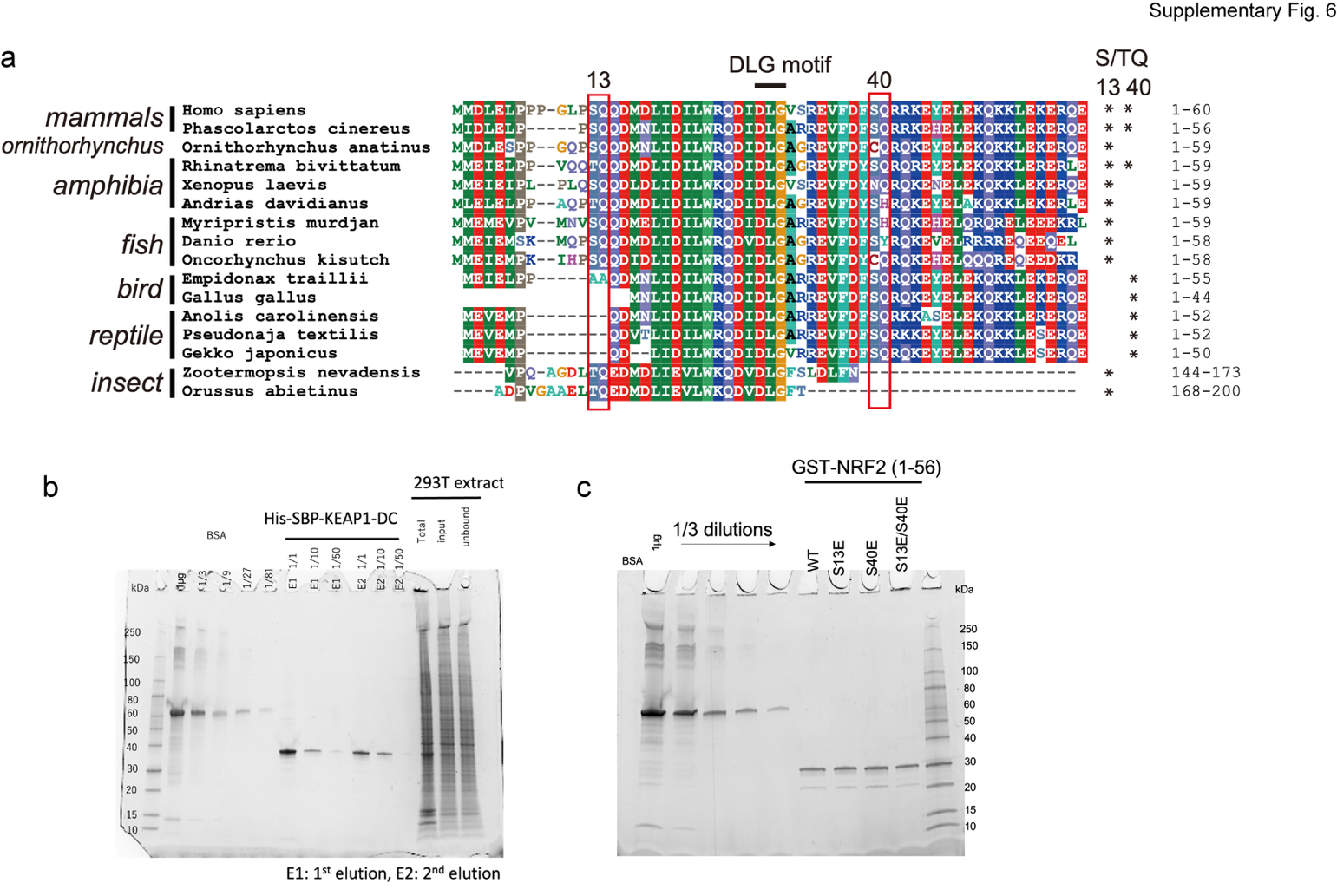
Supplementary Fig. 6**

Supporting data related to Fig. 5. **a** Sequence conservation in the N-terminal domain of NRF2. Multiple sequence alignments from selected species are shown. S/TQ motif is evolutionarily conserved (*). **b, c** Purification of recombinant His-SBP-KEAP1-DC (**b**) and GST-NRF2 (residues 1–56) (**c**) proteins used in Microscale thermophoresis analysis (Fig. 5b), visualized by Oriole staining.

**
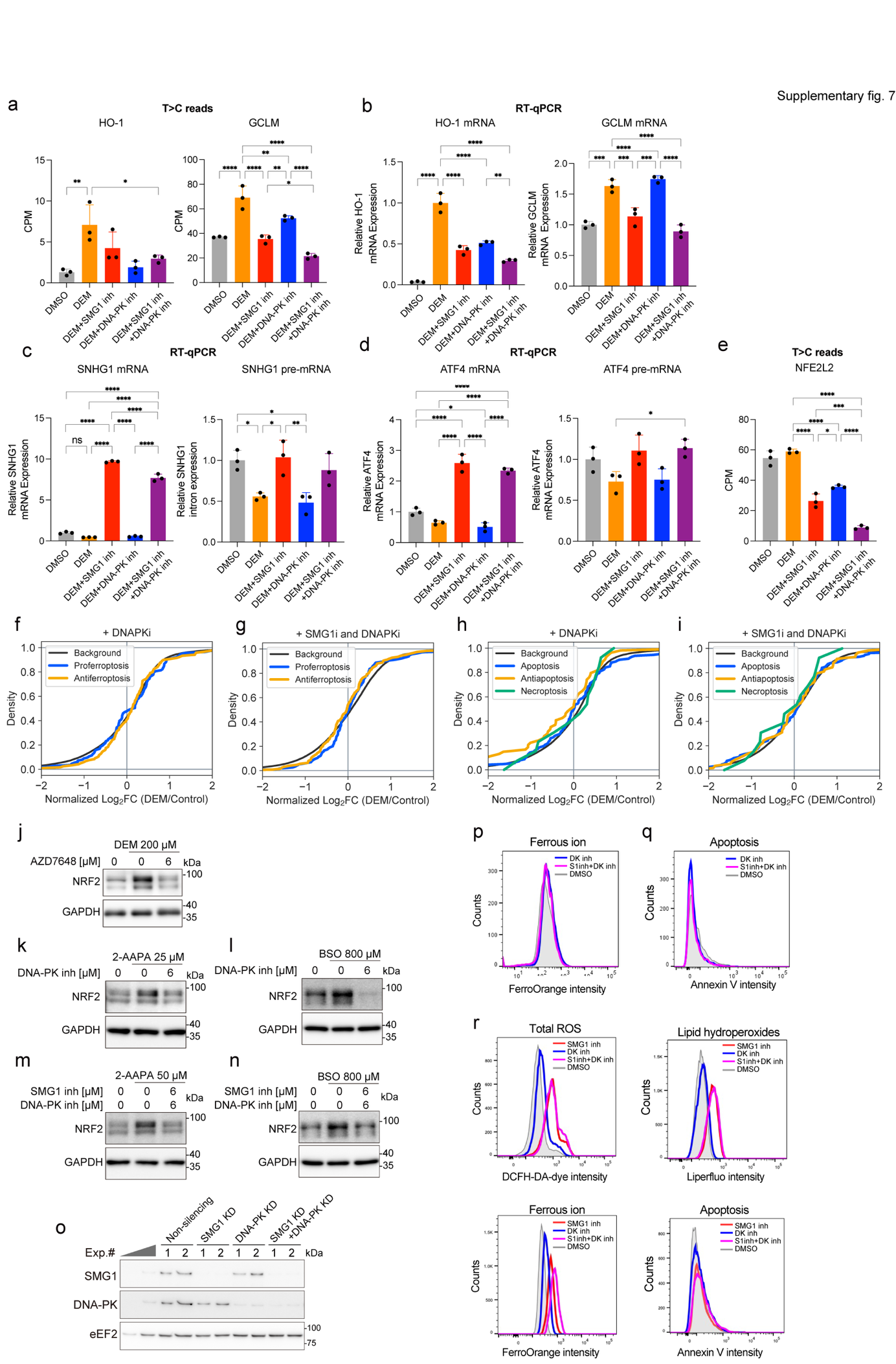
**

**Supplementary Fig. 7**

Supporting data related to Fig. 6, 7. **a** SLAMseq-derived counts per million (CPM) values of NRF2 target genes (HO-1 and GCLM) in PC-3 cells treated with DEM for 3 hours in the presence or absence of NU7441 or NU7441 plus NPD15008. **b** Relative mRNA levels of NRF2 target genes (HO-1 and GCLM) measured by RT–qPCR and normalized to 18S rRNA in PC-3 cells treated with 200 μM DEM in the presence or absence of NU7441 or NU7441 plus NPD15008 for 4 hours. **c**, **d** Relative mRNA levels of spliced mRNAs and introns of selected NMD targets, SNHG1 (**c**) and ATF4 (**d**) in PC-3 cells treated with 200 μM DEM with or without 4 μM NU7441 or 4 μM NU7441 plus 6 μM NPD15008 for 4 hours were determined by RT-qPCR, and normalized to 18S ribosomal RNA. Data were obtained through three independent experiments and values represent the means ± standard error. Statistical analysis was performed using one-way ANOVA with Tukey’s multiple-comparison test. *P<0.05, **P<0.01, ***P<0.001, ****P<0.0001. Experiments were performed with Figure S5B-D; therefore, identical DMSO, DEM, and DEM + SMG1 inhibitor datasets were used (**a**–**d**). **e** Counts per million (CPM) values of NRF2 in the SLAMseq in PC-3 cells treated with DEM in the presence of 4 μM NU7441 or 4 μM NU7441 plus 9 μM NPD15008. **f**, **g** Cumulative distribution of log₂ fold changes in de novo transcribed mRNAs for selected gene sets involved in pro-ferroptosis (blue) and anti-ferroptosis (orange) pathways, relative to all expressed genes (black). PC-3 cells were treated with 200 μM DEM in the presence of 4 µM NU7441 (**f**) or 4 µM NU7441 plus 9 μM NPD15008 (**g**) for 3 hours. Statistical significance was assessed using adjusted Welch’s t-tests. Adjusted Welch's t-test P-values: pro-ferroptosis genes (*P* = 1.0 in (**f**) and *P* = 1.0 in (**g**)), and anti-ferroptosis genes (*P* = 0.23 in (**f**) and *P* = 1.0 in (**g**)). Experiments were performed together with those shown in Supplementary Fig. 5**e**–**h**; therefore, identical DMSO, DEM, and DEM + SMG1 inhibitor datasets were used for comparative analysis. **h**, **i** Cumulative distribution of log₂ fold changes in de novo transcribed mRNAs for selected gene sets involved in pro-apoptosis (blue), anti-apoptosis (orange), and necroptosis (green) pathways, relative to all expressed genes (black). Statistical significance was assessed using adjusted Welch’s t-tests. Adjusted Welch's t-test P-values: pro-apoptosis genes (*P* = 1.0 in (**h**) and *P* = 1.0 in (**i**)), anti-apoptosis genes (*P* = 0.10 in (**h**) and *P* = 1.0 in (**i**)), and necroptosis genes (*P* = 1.0 in (**h**) and *P* = 1.0 in (**i**)). **j** Effect of AZD7648 on DEM-induced NRF2 accumulation. PC-3 cells were treated with 200 µM DEM with or without 6 µM AZD7648. **k**–**n** Effect of NU7441, alone or combined with NPD15008, on 2-AAPA or BSO-induced NRF2 accumulation. PC-3 cells were treated with 25 µM 2-AAPA or 800 µM BSO with or without 4 µM NU7441 (**k**, **l**) or 4 µM NU7441 plus NPD15008 (**m**, **n**) for 4 hours. Total cell extracts were probed with the antibodies indicated. GAPDH was probed as a loading control. The blots are representative of four independent experiments. **o** Representative results of siRNA-mediated knockdown of SMG1 and DNA-PK for Fig. 2g, h and 7a, b. PC-3 cells were transfected with the indicated siRNAs. Forty-eight hours after transfection, total cell lysates were analyzed by immunoblotting with the indicated antibodies. To estimate protein abundance, 33% and 11% of the non-silencing control samples were loaded. **p**, **q** Flow cytometric analysis of PC-3 cells following 24-hour treatment with 4 μM NU7441 or 4 μM NU7441 plus 6 μM NPD15008. Intracellular ferrous iron levels measured using the FerroOrange probe (**p**). Apoptosis assessed by annexin V staining (**q**). **r** Flow cytometric analysis of HepG2 cells following 24-hour treatment with 4 μM NPD15008, 7 μM NU7441, the combination of 7 μM NU7441 and 4 μM NPD15008, or 50 μM DEM. Intracellular total ROS, lipid hydroperoxide, ferrous iron, and apoptosis were assessed using DCFH-DA, Liperfluo, and FerroOrange probes, and annexin V staining, respectively.

**
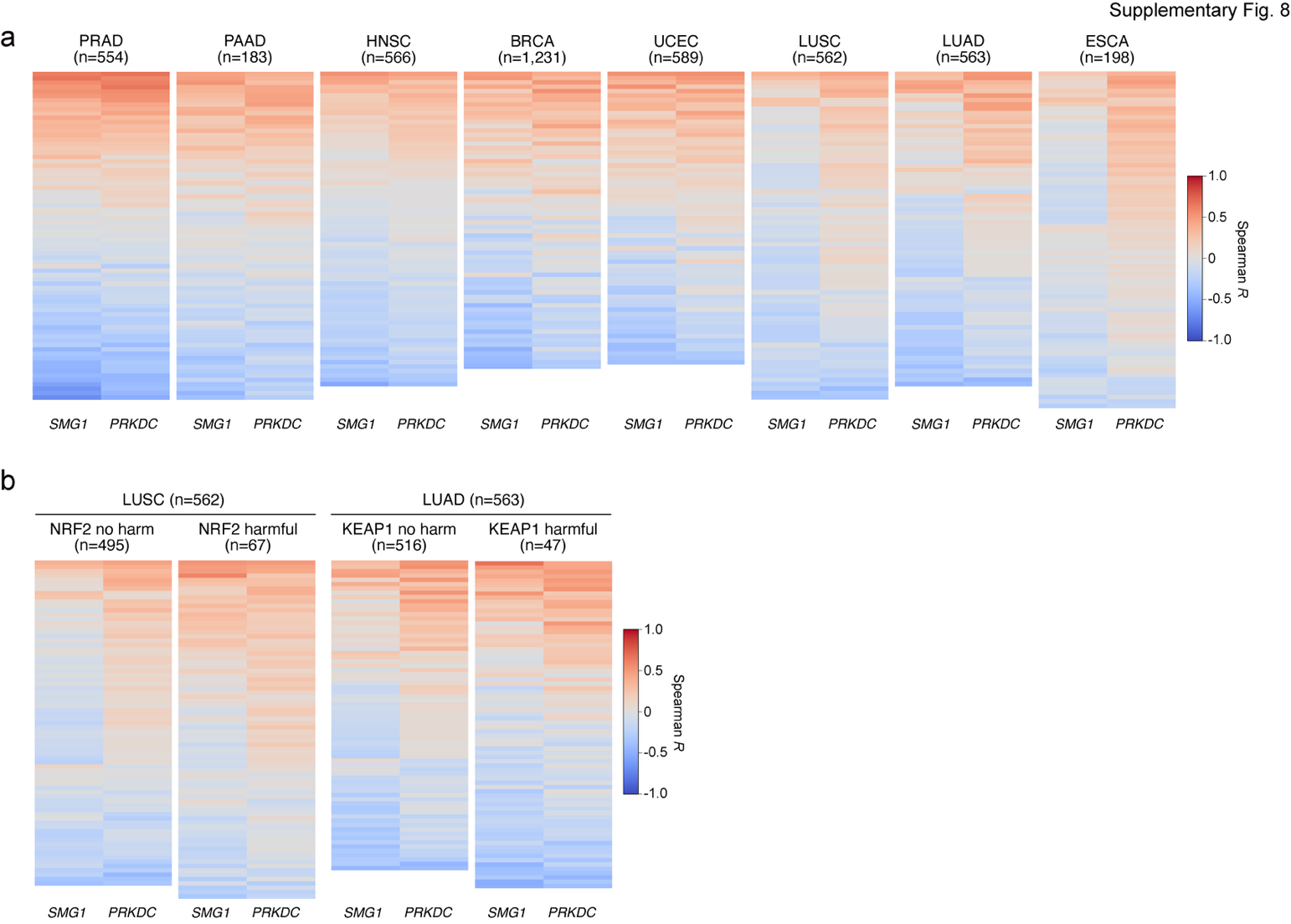
Supplementary Fig. 8**

Spearman correlation analysis of SMG1 and DNA-PKcs expression with NRF2 transcriptional activity across TCGA cancer cohorts. **a** Spearman correlation coefficients between 87 NRF2 target genes and *SMG1* or *PRKDC* (DNA-PKcs) based on TCGA RNA-seq datasets (PRAD, n = 554; PAAD, n = 183; HNSC, n = 566; BRCA, n = 1231; UCEC, n = 589; LUSC, n = 562; LUAD, n = 563; ESCA, n = 198). **b** Spearman correlation coefficients between 87 NRF2 target genes and *SMG1* or *PRKDC* based on TCGA -LUSC and TCGA-LUAD RNA-seq datasets. Samples were stratified by the presence of oncogenic mutations in *NFE2L2* (NRF2) (LUSC: harmful, n = 67; no harm, n = 495) or *KEAP1* (LUAD: harmful, n = 47; no harm, n = 516).

**Supplementary** **Table 1**

IC_50_ values of inhibition of PIKKs activity of 11 compounds using γ-^32^P-ATP. Details are described in materials and methods.

**Supplementary** **Table 2**

Summary results of the KINOMEscan. Values indicate the relative recovery of each kinases using ATP-beads in the presence of 10 μM compounds (e.g. 100: no effect; 10: 90% repression). Details are described in reference ^2^.

**Supplementary** **Table 3**

RNA-seq counts data for each condition.

**Supplementary** **Table 4**

RNA-seq data used for differential expression analysis.

**References**

1. Chen, J.*, et al.* Cell Death Triggers Induce MLKL Cleavage in Multiple Myeloma Cells, Which may Promote Cell Death. *Front Oncol* **12**, 907036 (2022).

2. Goldstein, D.M., Gray, N.S. & Zarrinkar, P.P. High-throughput kinase profiling as a platform for drug discovery. *Nat Rev Drug Discov* **7**, 391–397 (2008).
