# Supplementary material for: Two stress-responsive kinases suppress ferroptosis by activating antioxidant programs under mild oxidative stress
Source: Signal Transduct Target Ther. 2026 Aug 3;11:306. doi: 10.1038/s41392-026-02892-1 (PMC13429692; doi:10.1038/s41392-026-02892-1)
Supplement: Supplementary file 5 — Supplementary Table 1 [file 41392_2026_2892_MOESM5_ESM.docx]

**Supplementary Table 1 IC50 values of inhibition of PIKKs activity from 11 compounds identified from clinical development molecules using γ-32P-ATP and their specific substrate peptides.**

|  | SMG-1 | | | DNA-PK | ATR | mTOR | | ATM |
| --- | --- | --- | --- | --- | --- | --- | --- | --- |
| ATP [µM] | AlphaScreens | 10 | 1 | 10 | 1 | 10 | 1 | 1 |
| NPD15008 | 0.24 | 1.26 | 0.19 | > 50 | > 50 | > 50 | > 50 | > 50 |
| NPD12823 | 0.05 | 7.28 | 2.1 | > 50 | > 50 | > 50 | > 50 | > 50 |
| NPD12929 | 0.11 | 36.2 | 6.0 | 38.1 | 0.32 | - | 5.2 | 16.2 |
| NPD7884 | 0.32 | 34.7 | > 50 | 6.2 | > 50 | - | 19.8 | 6.7 |
| NPD13486 | 0.40 | 32.1 | > 50 | > 50 | > 50 | > 50 | 31.7 | > 50 |
| NPD14897 | 0.40 | 30.9 | 10.4 | > 50 | > 50 | > 50 | > 50 | > 50 |
| NPD11134 | 0.76 | 20.8 | 6.3 | > 50 | > 50 | > 50 | > 50 | > 50 |
| NPD8380 | 0.82 | 14.4 | 16.4 | > 50 | > 50 | - | 3.9 | 33.7 |
| NPD8625 | 0.88 | 21.8 | 15.8 | 3.5 | > 50 | 8.7 | 12.5 | 1.8 |
| CGS015943 | 0.014 | 0.58 | - | 6.6 | > 50 | - | 44.8 | 5.3 |
| GW0843682X | 0.387 | 3.2 | - | > 50 | > 50 | - | > 50 | 2.5 |
